# *Klebsiella pneumoniae* evolution in the gut leads to spontaneous capsule loss and decreased virulence potential

**DOI:** 10.1128/mbio.02362-24

**Published:** 2025-03-31

**Authors:** Lavinia V. Unverdorben, Ali Pirani, Kyle Gontjes, Bridget Moricz, Caitlyn L. Holmes, Evan S. Snitkin, Michael A. Bachman

**Affiliations:** 1 Department of Microbiology and Immunology, University of Michigan Medical School, Ann Arbor, Michigan, USA; 2 Department of Pathology, University of Michigan Medical School, Ann Arbor, Michigan, USA; National University of Singapore, Singapore, Singapore

**Keywords:** *Klebsiella*, intestinal colonization, capsule, evolution, mobile genetic elements, lipopolysaccharide

## Abstract

*Klebsiella pneumoniae* (*Kp*) is an opportunistic pathogen that poses a major threat in healthcare settings. The gut is a primary *Kp* reservoir in hospitalized patients, and colonization is a major risk factor for *Kp* infection. The stability of virulence determinants such as capsule and lipopolysaccharide during gut colonization is largely unexplored. In a murine gut colonization model, we demonstrated that spontaneous capsule loss occurs rapidly but varies by *Kp* pathotype. A classical *Kp* strain and a carbapenem-resistant strain of the epidemic sequence type 258 lineage had significant levels (median of 25% and 9.5%, respectively) of capsule loss. In contrast, a hypervirulent strain did not lose capsule to a significant degree (median 0.1%), despite readily losing capsule during *in vitro* passage. Insertion sequences (ISs) or mutations were found disrupting capsule operon genes of all isolates and in O-antigen encoding genes in a subset of isolates. Mouse-derived acapsular isolates from two pathotypes had significant fitness defects in a murine pneumonia model. Removal of the IS in the capsule operon in a mouse-derived acapsular classical isolate restored capsule production to wild-type levels. Genomic analysis of *Klebsiella* rectal isolates from hospitalized patients found that 18 of 245 strains (7%) had at least one IS disrupting the capsule operon. Combined, these data indicate that *Kp* capsule loss can occur during gut colonization in a strain-dependent manner, not only impacting strain virulence but also potentially altering patient infection risk.

**IMPORTANCE:**

In hospitalized patients, gut colonization by the bacterial pathogen *Klebsiella pneumoniae* (*Kp*) is a major risk factor for the development of infections. The genome of *Kp* varies across isolates, and the presence of certain virulence genes is associated with the ability to progress from colonization to infection. Here, we identified that virulence genes encoding capsule and lipopolysaccharide, which normally protect bacteria from the immune system, are disrupted by mutations during murine gut colonization. These mutations occurred frequently in some isolates of *Kp* but not others, and these virulence gene mutants from the gut were defective in causing infections. An analysis of 245 human gut isolates demonstrated that this capsule loss also occurred in patients. This work highlights that mutations that decrease virulence occur during gut colonization, the propensity for these mutations differs by isolate, and that stability of virulence genes is important to consider when assessing infection risk in patients.

## INTRODUCTION

*Klebsiella pneumoniae* (*Kp*) is a gram-negative bacterium and a leading cause of hospital-acquired infections in part due to its increasing antibiotic resistance (1, 2). *Klebsiella* species are the third most common cause of hospital-acquired infections in the United States, accounting for over 9% of these infections (3–5). Many infections are caused by classical *Kp* strains that are associated with multi-drug resistance and primarily cause infections in susceptible populations (6, 7). One classical pathotype of concern is the sequence type (ST) 258, which is carbapenem resistant through a plasmid-encoded *bla*_KPC_ carbapenemase. ST258 strains have led to global *Kp* outbreaks and are a major contributor to the distribution of *Kp* carbapenem resistance (8–10). In contrast, hypervirulent *Kp* strains can cause infections in both healthy and immunocompromised populations but tend to be more antibiotic susceptible (11, 12). These *Kp* pathotypes, and their convergence, have become a significant problem in healthcare settings (13).

One of the primary sites for *Kp* colonization is the gut, with colonization prevalence reported between 6 and 23% (14). *Kp* gut colonization increases the risk of extraintestinal *Kp* infection, as hospitalized patients with high (>22%) *Kp* gut abundance were three times more likely to develop an infection than patients with lower abundance (15). In nearly 80% of infected patients, the gut-colonizing strain was the same strain that caused a subsequent infection (16, 17). As colonization in the gut is associated with infection, it is critical to understand the *Klebsiella* factors that mediate colonization and subsequent infection.

Two well-established *Kp* virulence factors are the bacterial capsule and lipopolysaccharide (LPS), protecting *Kp* against phagocytosis by immune cells and complement-mediated serum killing (18–24). In sites of infection such as the lung, both capsule and LPS are important for *Kp* virulence (23, 25–27). Although studies have attempted to establish the role of the capsule in *Kp* gut colonization, results have been inconsistent across strain backgrounds and gene mutations (28–30). In the SGH10 strain, an *rmpA* mutant has increased fitness, a *wcaJ* mutant has initial decreased fitness and then a fitness advantage, and *wza* and *wzy* mutants have fitness defects, whereas mutation of *manC* in Kp52145 impairs gut colonization (29–31). Despite being a virulence factor, capsule inactivation has been reported in *Kp* clinical isolates and in nutrient-rich *in vitro* conditions, suggesting that under certain conditions, the capsule may be dispensable (32–34). The environmental conditions that drive capsule loss and the potential implications of this on patient infection risk are unclear.

Insertion sequences (ISs), transposable elements that play a crucial role in genome plasticity and adaptability among bacteria, are one mechanism of capsule disruption (35–37). ISs are small (0.7 kb–2.5 kb), consisting of one to two open reading frames, and are common in bacterial chromosomes and plasmids. Bacteria can acquire ISs from other microbes through horizontal gene transfer, facilitating acquisition of new traits such as antibiotic resistance, metabolic adaptation, and capsule loss (38–41). In *Klebsiella*, transposition of ISs from plasmids has been associated with capsule inactivation, colistin resistance, and strain adaptation *in vitro* and *in vivo (*34, 35, 38, 42).

To investigate the prevalence and role of capsule inactivation during *Kp* gut colonization and infection, we used three different *Kp* strains: (i) Kp4819, a classical strain isolated from the gut of a hospitalized patient, (ii) NJST258_2, a ST258 classical carbapenem-resistant strain, and (iii) KPPR1, a hypervirulent strain. We determined that the classical *Kp* strain loses capsule readily and that ISs led to capsule inactivation. Despite high levels of capsule loss during *in vitro* passage, the hypervirulent KPPR1 strain did not display significant capsule loss during gut colonization. Evolved acapsular isolates from classical and hypervirulent pathotypes had a fitness disadvantage in a murine pneumonia model. Finally, an analysis of a cohort of *Klebsiella* rectal isolates from hospitalized patients demonstrated that capsule-disrupting ISs were prevalent in colonizing strains. These data suggest that *Kp* evolution leading to capsule loss in the gut is strain dependent and may impact virulence potential at extraintestinal sites.

## RESULTS

### *Klebsiella pneumoniae* spontaneous capsule loss occurs during gut colonization

**Fig 1:**
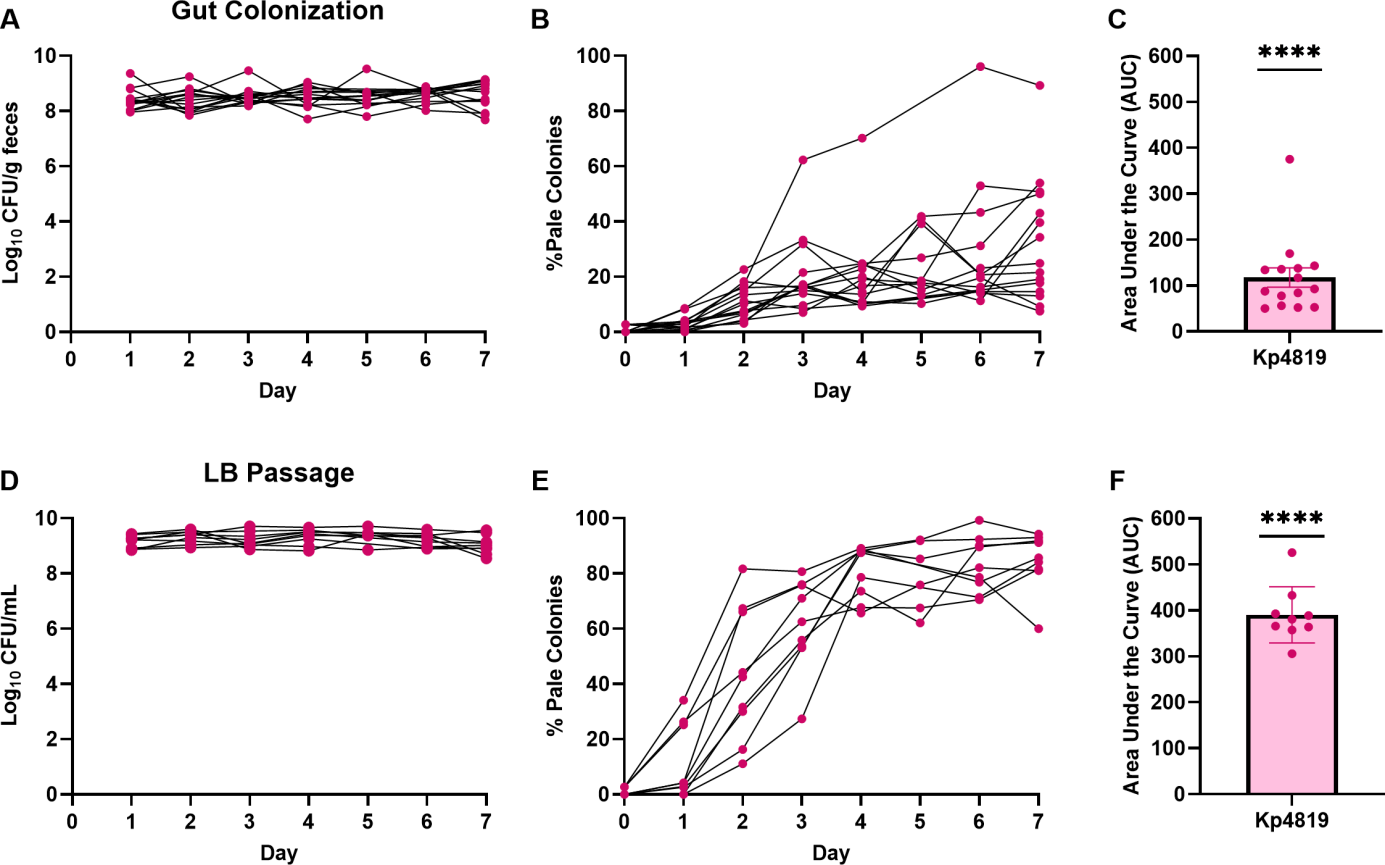
*K. pneumoniae* Kp4819 loses capsule during gut colonization and LB passage. Kp4819 *in vivo* capsule loss was determined using a murine gut colonization model (A–C) and *in vitro* during LB passage (D–F). Mice received 5 × 10^6^ CFU of Kp4819 via oral gavage, and 5 µL of the inoculum was also added to three tubes containing 5 mL of LB and passaged concurrently. For LB passaging, LB cultures were diluted 1:1,000 into 5 mL of fresh LB broth every 24 hours and an aliquot was plated to quantify total pale colonies. Kp4819 was quantified by culture and displayed as log_10_ CFU/g of feces (A) or as log_10_ CFU/mL (D). The percentage of pale colonies is displayed (B, E) and summarized as area under curve (C, F) with mean and SEM shown. For panels A–C, *n* = 15 across three independent experiments. For panels D–F, *n* = 9 across three independent experiments. For panels C and F, ****, *P* < 0.0001 by one-sample *t*-test with a hypothetical mean = 0.

To determine whether *Kp* capsule loss occurs during gut colonization, we used Kp4819, a classical strain isolated from the gut of a patient who developed a *Kp* urinary tract infection (Table 1) (17, 43). In a murine gut colonization model, antibiotic-treated mice were inoculated with Kp4819, and fecal sample culture was used to monitor colonization and pale colony development (a phenotype associated with capsule loss) (44). Kp4819 colonized mice at levels between 10^7^ and 10^9^ colony forming units (CFU)/g of feces across all 7 days of the experiment (Fig. 1A). Pale colonies developed in all colonized mice, with a median of 25% of the total population turning pale at day 7 (Fig. 1B), representing a significant accumulation of pale colonies (Fig. 1C). As a control, the inoculum was passaged concurrently in Luria-Bertani (LB) broth, a nutrient-rich condition which promotes spontaneous capsule loss (32). Growth of Kp4819 in LB was robust with Kp4819 reaching 10^8^–10^9^ CFU/mL (Fig. 1D), and significant pale colony development occurred with a median of 86% of the total population turning pale by day 7 (Fig. 1E and F).

**TABLE 1:** Summary of strains used in this study

Strain	Species	Isolation source	Sequence type	K type	O-serotype	Virulence score	Genome size (Mb)	Reference
Kp4819	*K. pneumoniae*	Gastrointestinal tract, human	ST1083	KL132	O1/O2v1	0	5.49	PMID: 35915063
KPPR1	*K. pneumoniae*	Rifampin-resistant isolate of ATCC43816	ST493	KL2	O1/O2v1	1 (*ybt*, *rmp*)	5.36	PMID: 33541885
NJST258_2	*K. pneumoniae*	Urinary tract, human	ST258	KL107	O1/O2v2	0	5.29	PMID: 24639510

**Fig 2:**
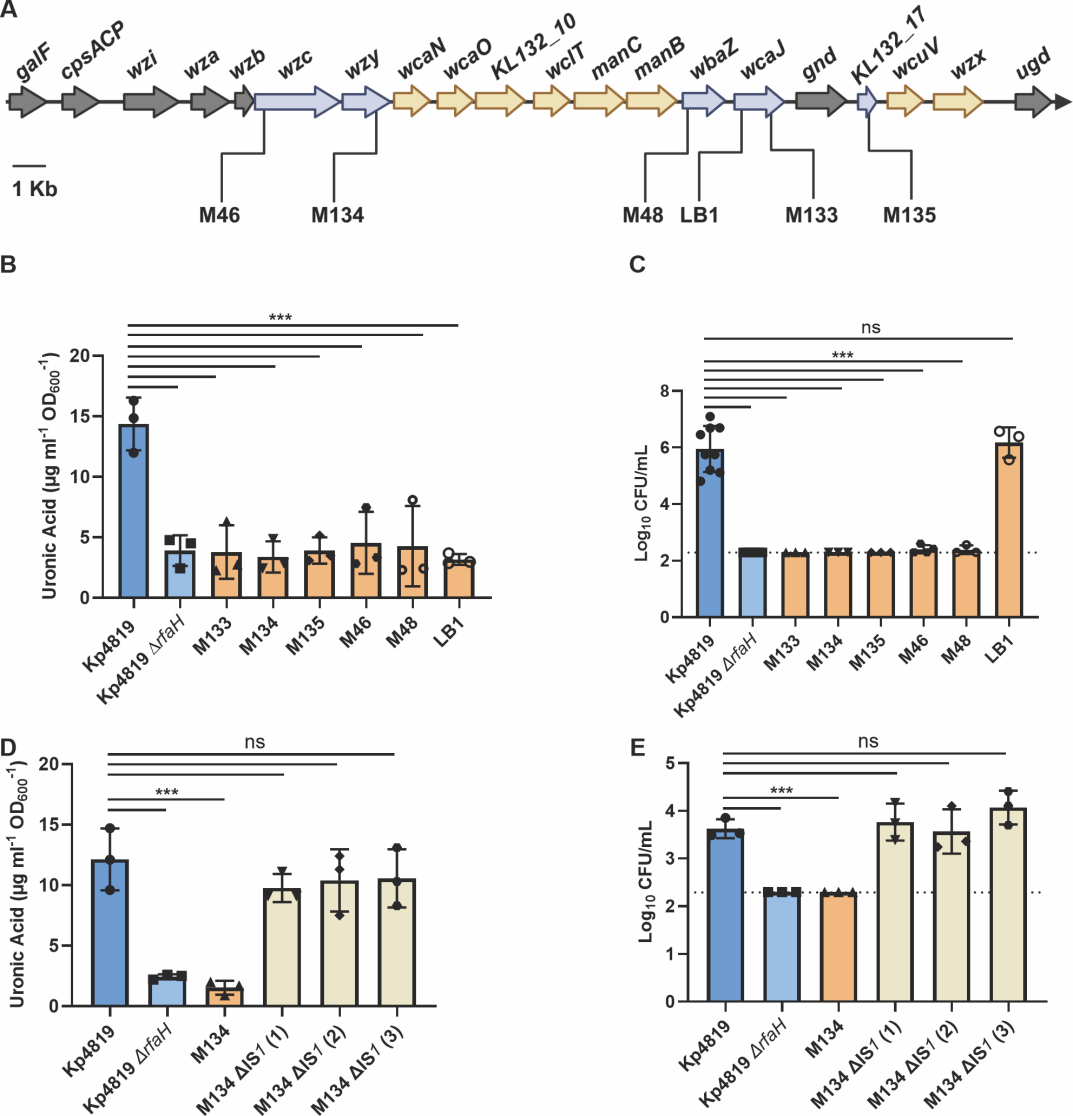
Spontaneous insertion sequences in the capsule operon lead to acapsular Kp4819 phenotypes. Mutations in the capsule operon of six acapsular Kp4819 isolates from mouse gut colonization (Kp4819_M46-M135) or LB passaging (Kp4819_LB1) were identified by whole genome and Sanger sequencing (A). Genes in gray represent the beginning and end of the capsule operon, genes with at least one disruption are in blue, while all other capsule genes are in yellow. Capsule production of Kp4819, Kp4819 ∆*rfaH*, and six Kp4819 pale isolates was determined via uronic acid quantification (B). Susceptibility to active human serum after 3 hours was compared for 1 × 10^5^ CFU of Kp4819, Kp4819 ∆*rfaH*, and six acapsular Kp4819 isolates with the acapsular Kp4819 ∆*rfaH* mutant serving as a control (C). Capsule production of Kp4819_M134 isolates with the IS in the capsule operon removed via allelic exchange (M134 ∆IS*1* 1–3) was determined via uronic acid quantification (D). Susceptibility to active human serum after 3 hours was compared for 1 × 10^5^ CFU of Kp4819, Kp4819 ∆*rfaH*, and three Kp4819_M134 clones with the IS in the capsule region removed (M134 ∆IS*1* 1–3) via allelic exchange (E). For panels B–E, statistical significance between the Kp4819 WT and each isolate was determined by one-way analysis of variance with *n* ≥ 3 with Dunnett’s multiple comparisons test with ***, *P* < 0.001.

We further characterized six Kp4819 pale colonies, five from *in vivo* gut colonization designated with the prefix M (Kp4819_M46, Kp4819_M48, Kp4819_M133-M135), and one from *in vitro* LB passaging (Kp4819_LB1). All strains except for Kp4819_M133 had similar growth as the Kp4819 wild-type (WT) in LB, but differences were observed in minimal media (Fig. S1). To determine whether these isolates had reduced capsule production compared to the WT, extracellular uronic acids were quantified (45). All six isolates had a significant decrease in capsule production compared to the WT and produced similar amounts of uronic acid as the acapsular Kp4819 ∆*rfaH* mutant (25) (Fig. 2B). These results indicate that a clinical isolate such as Kp4819 has a high propensity for capsule loss during gut colonization.

### Insertion sequences in the capsule and O-antigen operons are found in acapsular KP4819 isolates

As previous work demonstrated that spontaneous capsule loss is associated with capsule gene inactivation, we performed whole genome sequencing of all six acapsular Kp4819 isolates (33, 34). Using the bioinformatic tool panISa and confirmation with Sanger sequencing, we found that an IS*1* family IS disrupted the capsule operon of all six isolates (46) (Fig. 2A, Table 2). Kp4819_LB1 and Kp4819_M133 had an IS in different parts of the glycosyltransferase gene, *wcaJ*. Kp4819_M134 had an IS in *wzy* encoding the wzy polysaccharide polymerase, while Kp4819_M135 had an IS in a gene predicted to encode a serine acetyltransferase. Kp4819_M46 had an IS in *wzc*, encoding a tyrosine protein kinase, and Kp4819_M48 in *wbaZ,* a gene encoding a group 1 glycosyl transferase.

**TABLE 2:** *K. pneumoniae* spontaneous capsule mutants from gut colonization and LB passaging

Isolate	Isolation environment	Capsule-related mutations (gene [mutation]; nucleotide location)	Capsule-relateddeletion events	O-antigen mutation (gene [mutation]; nucleotide location)	O-antigen-related deletion events	Additional mutations (gene [mutation]; nucleotide location)
Kp4819_M133	Mouse	*wcaJ* (IS*1X2*); 1734621		*wbbM* (T → TG); 1748628		
Kp4819_M134	Mouse	*wzy* (IS*1X2*); 1725391		*wbbM* (IS*1X2*); 1749043		
Kp4819_M135	Mouse	*KL*132_17 (IS*1X2*); 1737565		*wbbN* (IS*Kpn74*); 1751657		GNCKPDFN_02192–02193 (IS*Kpn49*); 2244895 GNCKPDFN_02570 (IS*Kpn63*); 2611751 *ibpB_2* (IS*1X2*); 2706030
Kp4819_M46	Mouse	*wzc* (IS*1X2*); 1722143				GNCKPDFN_02570 (IS*Kpn63*); 2611751
Kp4819_M48	LB	*wbaZ* (IS*1X2*); 1733180				GNCKPDFN_02192–02193 (IS*Kpn49*); 2244895 GNCKPDFN_02570 (IS*Kpn63*); 2611751 *ecpC* (IS*903*); 4170546
Kp4819_LB1	Mouse	*wcaJ* (IS*1X2*); 1734525				GNCKPDFN_02192–02193 (IS*Kpn49*); 2244895 GNCKPDFN_02570 (IS*Kpn63*); 2611751
KPPR1_M287	Mouse	*rfaH* (G → C); 3218876	5178936–5179143 (VK055_RS25400); 208 bp			
KPPR1_M288	Mouse		5173021–5197360 (VK055_RS29345); 24,340 bp		5197491–5203585 (VK055_RS25495); 6,095 bp 5203880–5212762 (VK055_RS25495 - VK055_RS25540); 8,883 bp	
KPPR1_M289	Mouse	*rfaH* (C→ T); 3218710				
NJST258_2_M330	Mouse	*wzc* (IS*Kpn26*); 1801262				KPNJ2_RS20540 (G → A); 4068593
NJST258_2_M331	Mouse	*wzc* (IS*Kpn26*); 1801718	5056689–5056848 (upstream of *rfaH*); 160 bp			*rsxC* (G → T); 2398856 KPNJ2_RS20540 (C → CAGGTCG); 4068055
NJST258_2_M357	Mouse					

As studies have shown a connection between capsule production and LPS, we also looked for ISs or mutations in the *rfb* operon encoding O-antigen (47, 48). Three out of the six Kp4819 isolates had additional ISs or mutations in this operon (Table 2). Kp4819_M134 had the same IS*1* family IS in *wbbM* as in the capsule operon, while Kp4819_M135 had an IS*5* family IS*903* in the glycosyltransferase *wbbN*. Kp4819_M133 had a single nucleotide insertion in the *wbbM* gene, which led to a frameshift mutation. The remaining acapsular isolates had no mutations or variants in the *rfb* operon or other known LPS biosynthesis genes. Additional IS events and mutations outside of the capsule and O-antigen operons were observed in Kp4819_M135, M46, M48, and LB1 (Table 2).

### Spontaneous capsule loss increases Kp4819 susceptibility to human serum killing

Given that the capsule is required for *Klebsiella* resistance to killing by active human serum*,* we investigated whether lack of capsule biosynthesis conferred serum susceptibility. All acapsular Kp4819 isolates from *in vivo* gut colonization experiments (Kp4819_M46-M135) were susceptible to killing by active human serum (Fig. 2C). However, the acapsular isolate Kp4819_LB1 from *in vitro* passaging was serum resistant despite having a significant decrease in capsule production (Fig. 2B). Two additional Kp4819 isolates from LB passaging were also acapsular and serum resistant (Fig. S2A and B). Although only three Kp4819 LB isolates were tested, this suggests that the type of environment where capsule loss develops, or the nature of capsule inactivation, may play a role in *Klebsiella* serum susceptibility.

### Evolved KP4819 isolates display differences in LPS structure

**Fig 3:**
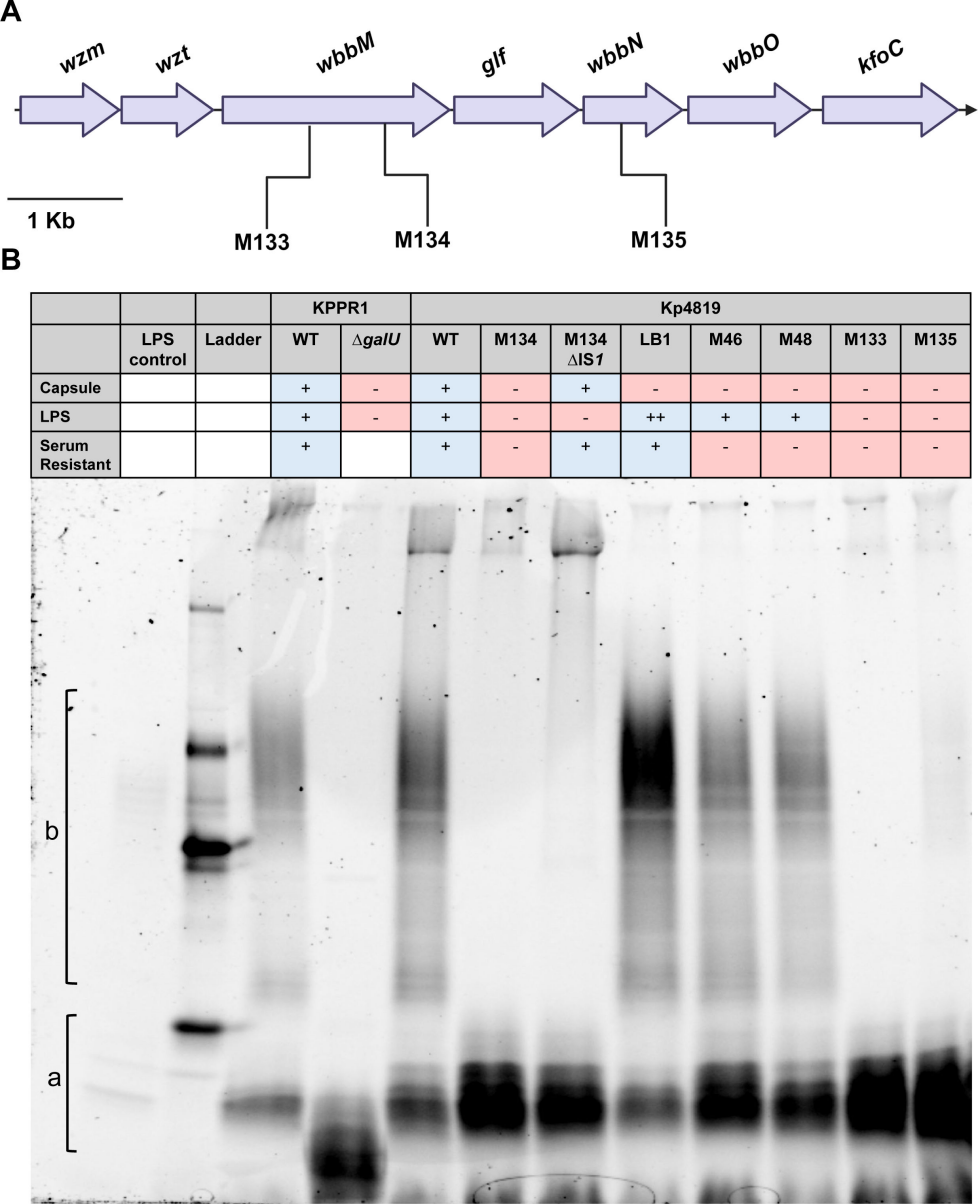
Evolved *K. pneumoniae* Kp4819 acapsular isolates have differing LPS structures. Insertion sequences found in the O-antigen encoding *rfb* operon of acapsular Kp4819 isolates were identified by whole genome sequencing (A). LPS was isolated from 1 × 10^9^ CFU of *Kp* strains of interest, and 10 µL of each LPS product was visualized using SDS-PAGE (B). An *Escherichia coli* LPS control is the first lane, followed by the CandyCane molecular weight ladder, and the *Kp* isolates of interest. The LPS core region is labeled a while the O-antigen region is labeled b.

To determine the impact of the ISs and mutations on LPS structure (Fig. 3A; Table 2), LPS was isolated and visualized using SDS-PAGE (Fig. 3B). The KPPR1 WT and a previously characterized isogenic ∆*galU* mutant were used as controls that have intact O-antigen and capsule or lack them, respectively (49). The Kp4819 WT had an intact LPS structure with visible core and O-antigen polysaccharides. The Kp4819_M133, M134, and M135 isolates had no visible O-antigen laddering most likely due to the mutations in the *wbbM* and *wbbN* genes, respectively (Table 2). Kp4819_M46 and Kp4819_M48, two isolates with no *rfb* operon or LPS mutations, had similar intact core and O-antigen structures (Fig. 3B). Interestingly, acapsular Kp4819_LB1, which was serum resistant, displayed a unique LPS O-antigen banding pattern. This suggests that the serum resistance of Kp4819_LB1 may be related to differences in O-antigen structure such as polysaccharide chain length or increased polysaccharide quantity that compensates for capsule loss. These data highlight that evolution during gut colonization leads to changes in O-antigen as well as capsule.

### IS*1* insertion in *wzy* is sufficient to cause capsule loss

For subsequent experiments, Kp4819_M134 was chosen for characterization as it was the only isolate without growth defects or potentially confounding mutations outside of capsule and O-antigen operons. To determine whether removal of ISs located in the capsule operon was sufficient to restore capsule production, single gene complementation of *wzy* in Kp4819_M134 was attempted but did not restore WT capsule levels (Fig. S2C). Instead, we leveraged allelic exchange using lambda red-mediated homologous recombination to remove the IS disrupting the *wzy* gene in Kp4819_M134, replacing it with the Kp4819 WT sequence and selecting for successful capsular transformants in human serum (50). Successful removal of the IS was confirmed via PCR and opaque colony morphology. Uronic acid quantification of three Kp4819_M134 clones with the IS removed (Kp4819_M134 ∆IS*1* 1–3) demonstrated that IS removal restored capsule production to WT levels (Fig. 2D). All clones had restored resistance to active human serum (Fig. 2E). As expected, removal of the IS in the Kp4819_M134 capsule operon (Kp4819_M134 ∆IS*1*) did not restore O-antigen production (Fig. 3B). To examine the stability of the Kp4819_M134 acapsular phenotype during *in vitro* passage and gut colonization, mice and LB broth samples were inoculated in parallel with Kp4819_M134. Consistent with the expectation that spontaneous excision of the IS element is unlikely to restore the wild-type gene sequences, there was no detectable reversion of pale colonies to WT during LB passage. During gut colonization, Kp4819_M134 colonized at similar levels as the WT, and minimal reversion to the WT was observed with only 1% of opaque colonies observed on day 7 (Fig. S2 D through I). These data demonstrate that IS*1* family ISs are sufficient for stable capsule disruption and occur readily during Kp4819 gut colonization.

### Gut-evolved isolate Kp4819_M134 has reduced fitness at extraintestinal sites

**Fig 4:**
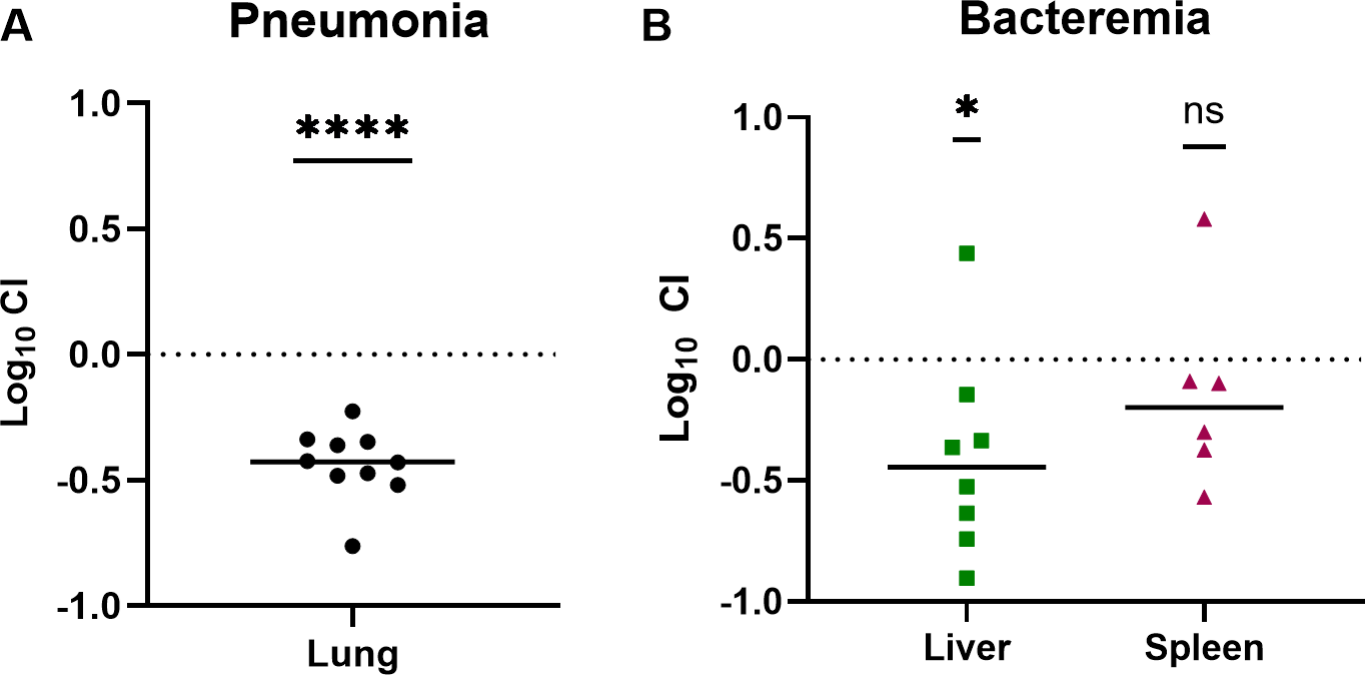
An evolved acapsular Kp4819 isolate has fitness defects during models of infection. Kp4819_Kan_ was competed against the acapsular Kp4819_M134 isolate in a murine pneumonia (A) or bacteremia (B) model. In a murine pneumonia model, mice were inoculated with 1 × 10^8^ CFU of a 1:1 mixture of Kp4819_Kan_ and Kp4819_M134 retropharyngeally (A). To model murine bacteremia, mice were administered 1 × 10^7^ CFU of a 1:1 mixture of Kp4819_Kan_ and Kp4819_M134 via tail vein injection (B). The mean log_10_ competitive index at 24 hours post-infection is displayed for both panels A and B. For panels A and B, *, *P* < 0.05 and ****, *P* < 0.0001, by one sample *t*-test and *n* ≥ 8 performed across two independent experiments.

To determine whether an acapsular Kp4819 strain was attenuated, Kp4819_M134 was competed against kanamycin-marked Kp4819 (Kp4819_Kan_), which has equivalent growth and capsule loss kinetics *in vitro* as Kp4819 (Figs. S3 and 4). In a gut colonization model, Kp4819_M134 had no fitness defect compared to Kp4819_Kan_ on days 1–4 and 7 of gut colonization, with a mild defect on days 5–6 (Fig. S5). In this competition with an acapsular strain, there was little capsule loss of Kp4819_Kan_ (Fig. S5). In a murine pneumonia model, Kp4819_M134 had an approximately threefold fitness defect compared to Kp4819_Kan_ (Fig. 4A; Fig. S6A). In a murine model of bacteremia, Kp4819_M134 had a threefold fitness defect in the liver compared to Kp4819_Kan_ but did not have a fitness defect in the spleen (Fig. 4B; Fig. S6B). Together, these data suggest that Kp4819 capsule loss during gut colonization reduces virulence at specific extraintestinal sites during infection.

### *Klebsiella* pathotypes show variability in spontaneous capsule loss during gut colonization

**Fig 5:**
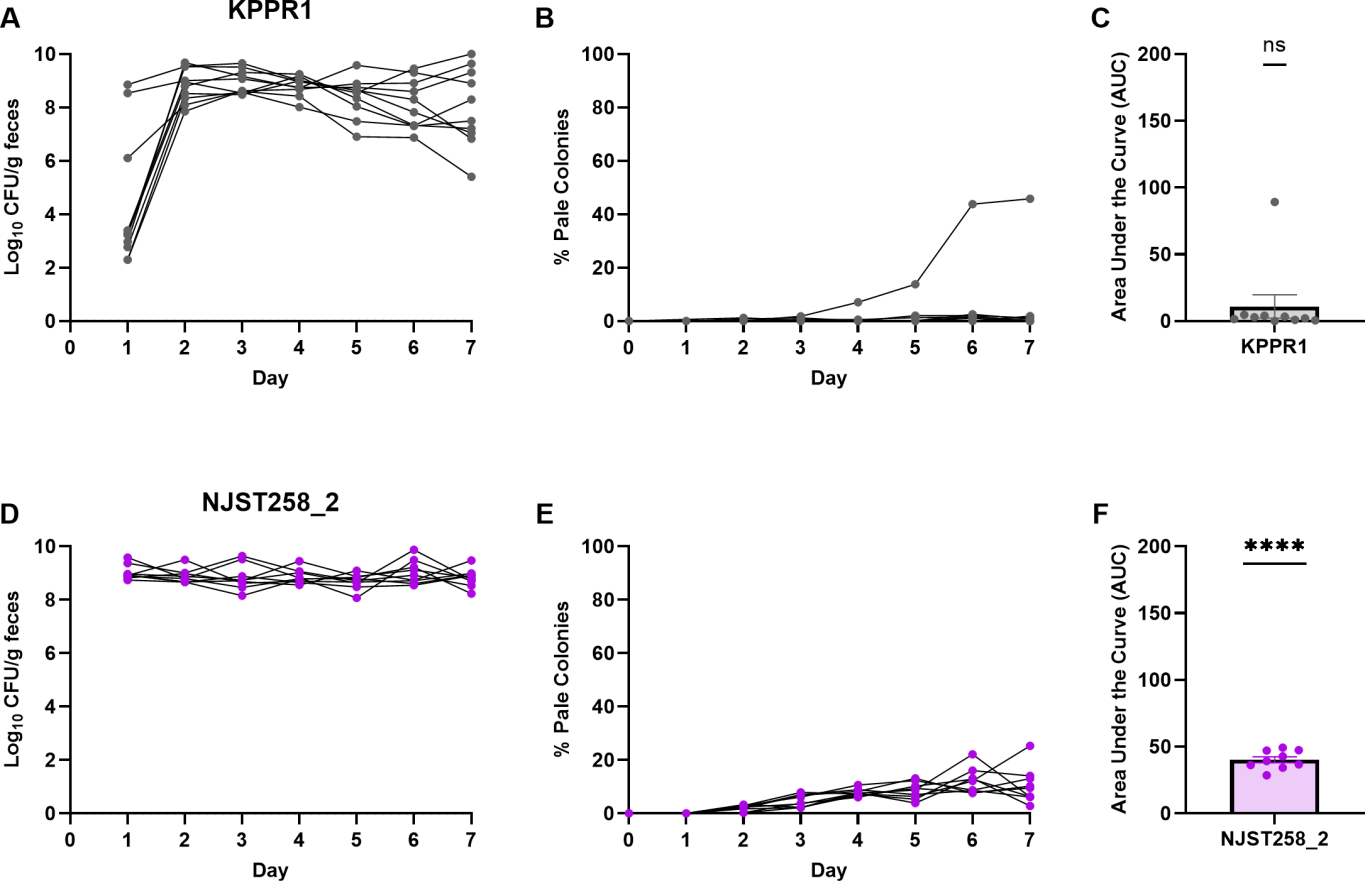
*K. pneumoniae* pathotypes vary in spontaneous capsule loss during gut colonization. In a murine gut colonization model, mice were inoculated with 5 × 10^6^ CFU of KPPR1 (A–C) or NJST258_2 (D–F) via oral gavage. The levels of *Kp* in the gut were measured by fecal collection and displayed as log_10_ CFU/g feces (A, D). The development of pale colonies was monitored for each strain during gut colonization and displayed as percentages (B, E) and as area under the curve (C, F) with mean and SEM. For panels C and F, ****, *P* < 0.0001 by one sample *t*-test with a hypothetical mean = 0. For panels A–F, *n* ≥ 9 across two independent experiments.

To determine whether spontaneous capsule loss during gut colonization is generalizable, we investigated whether different *Kp* pathotypes displayed capsule loss during gut colonization. The hypervirulent strain KPPR1 and NJST258_2, a *bla*_KPC_ carbapenemase-producing ST258 isolate, colonized the mice at consistently high levels except for KPPR1 on day 1, in which colonization levels ranged 10^2^–10^8^ CFU/g feces (Fig. 5A and D). Although KPPR1 readily lost capsule during passage in LB (Fig. S7A through C), there was not a statistically significant accumulation of acapsular colonies during colonization (Fig. 5B and C). Except for one outlier, a median of 0.1% acapsular colonies was recovered on day 7 during KPPR1 colonization (Fig. 5B). Across all mice, NJST258_2 had significant capsule loss with a median of 9.5% acapsular colonies recovered by day 7 (Fig. 5E and F). Growth curve assays and uronic acid quantification of three KPPR1 and NJST258_2 pale isolates from three different mice (KPPR1_M287-289 and NJST258_2_M330-331, NJST258_2_M357) were performed. All isolates did not have replication differences in LB, but differences were observed in M9+glucose (Fig. S8). Uronic acid quantification confirmed that evolved pale KPPR1 and NJST258_2 isolates were acapsular (Fig. S9A and C). All acapsular KPPR1 isolates were susceptible to killing by human serum (Fig. S9B). Serum killing for NJST258_2 isolates was not performed as NJST258_2 is susceptible to killing by human serum at concentrations greater than 25% (51). These data suggest that *Kp* pathotypes have differing propensity for capsule loss during colonization, with the hypervirulent KPPR1 strain retaining its capsule compared to the classical and carbapenemase-producing strains tested.

### KPPR1 and NJST258_2 acapsular isolates have diverse genetic changes

To determine the genetic mechanism underlying spontaneous capsule loss, we performed whole genome sequencing of KPPR1 and NJST258_2 acapsular isolates. Three KPPR1 isolates had differing genetic changes most likely responsible for capsule loss (Table 2). Both KPPR1_M287 and KPPR1_M289 had single nucleotide polymorphisms within or upstream of the capsule and LPS regulator *rfaH* and no changes in any LPS biosynthesis genes. KPPR1_M287, when competed in a murine pneumonia model, had a significant fitness defect in the lung compared to the KPPR1 WT, confirming decreased virulence of an additional acapsular isolate at extraintestinal sites (Fig. S10). The KPPR1_M288 isolate had significant deletion events in both the capsule and O-antigen operons (Table 2). For evolved NJST258_2 isolates, two out of three (NJST258_2_M330-M331) had an IS*Kpn26* disrupting the *wzc* tyrosine kinase in the capsule operon (Table 2). NJST258_2_M357 did not have any genetic changes in capsule or LPS biosynthesis genes but did have a deletion upstream of the capsule regulator *rfaH*. Both NJST258_2_M330 and M331 had additional mutations outside of the capsule and O-antigen operons (Table 2). These data indicate distinct genetic changes in evolved acapsular KPPR1 and NJST258_2 isolates compared to the IS*1X2* insertions observed with Kp4819.

### Anaerobic conditions limit spontaneous capsule loss across *Kp* pathotypes

**Fig 6:**
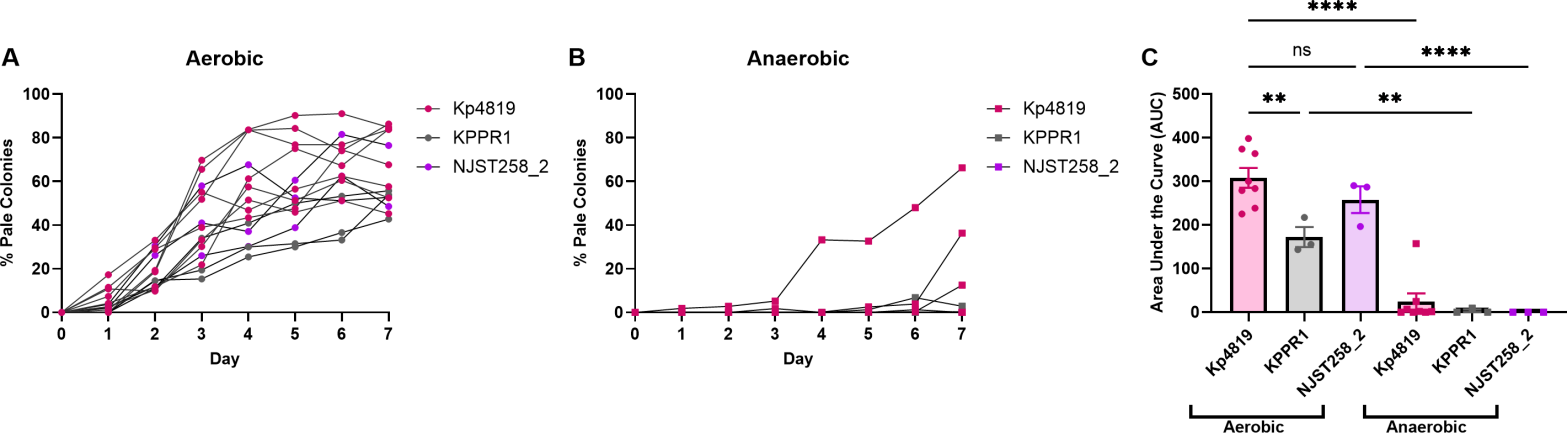
Anaerobic conditions inhibit spontaneous capsule loss across *K. pneumoniae* pathotypes. Kp4819, KPPR1, and NJST258_2 were passaged in 5 mL of LB in aerobic (A) or anaerobic conditions (B) to monitor for pale colony formation. Every 24 hours, LB cultures were diluted 1:1,000 into 5 mL of fresh LB broth, and an aliquot of each sample was plated to quantify total pale colonies. The percentage of pale colonies and (A, B) and area under the curve (C) with mean with SEM is displayed. For panel C, **, *P* < 0.01 and ****, *P* < 0.0001, by one-way analysis of variance with an *n* ≥ 3 and Šídák’s multiple comparisons test.

As the gut is an environment with differences in oxygen availability, we assessed the impact of oxygen availability on capsule loss. Kp4819, KPPR1, and NJST258_2 were passaged under aerobic and anaerobic conditions in LB broth. Although all three strains showed similar growth in aerobic conditions, growth was between a half and three-quarters log lower in anaerobic conditions (Fig. S11). By day 7 of aerobic passage in LB broth, a median of 58% acapsular Kp4819, 54% acapsular KPPR1, and 53% acapsular NJST258_2 colonies were recovered (Fig. 6A). Based on area under the curve (AUC) analysis, there was a significant difference in the kinetics between strains, with KPPR1 accumulating less acapsular colonies than Kp4819 (Fig. 6C). In anaerobic passage, all three strains developed significantly fewer acapsular colonies with median values of 12.5%, 0%, and 0% for Kp4819, KPPR1, and NJST258_2, respectively (Fig. 6B and C). This suggests that the combination of aerobic and nutrient-rich conditions promotes spontaneous capsule loss, whereas it is inhibited under anaerobic conditions.

### Acapsular isolates colonize hospitalized patients

To investigate the frequency and causes of capsule inactivation during human gut colonization, we searched for ISs in the capsule operon of diverse 245 *Klebsiella* rectal isolates from a previously published case/control study (Table S3) (17). Gut-colonizing *Klebsiella* rectal isolates were recovered from patients in intensive care and hematology/oncology units. Of these isolates, 18 had a minimum of one IS in the capsule operon as identified by panISa and ISFinder (Fig. S12, Table 3) (46, 52). Of these isolates, six were isolated from colonized patients who developed a subsequent *Klebsiella* infection (case). The 18 isolates represented 13 unique capsule types and 17 sequence types. The seven ISs identified belonged to three IS families: IS*1* (IS*Kpn14*, IS*1X2*, IS*Ec30*), IS*5* (IS*Kpn74*, IS*903B*), and IS*630* (IS*Ec33*, IS*Sen14*) (Table 3). IS*1X2* was the same ISs disrupting the capsule operon in acapsular Kp4819 isolates from the murine gut colonization experiments, and IS*Kpn*26 that disrupted NJST258_2 capsule *in vivo* is part of the IS*5* family that disrupted capsular operons in the rectal isolates. For five isolates, two predicted to and three not predicted to have an IS, we validated the bioinformatic analysis using PCR and Sanger sequencing with 100% concordance (data not shown). The presence of ISs in the capsule operon of rectal isolates from patients suggests that the mechanism of capsule loss observed *in vivo* in mice may also be of clinical importance and a factor when assessing patient infection risk.

**TABLE 3:** Human rectal swab isolates of *K. pneumoniae* with insertion sequences in capsule genes

Strain	Case status	Infection	Species	Sequence type	Clonal group type	K locus	IS element	Position
Kp4955	Control		Kp	ST268		K20	IS*Kpn74*	2078–2086
Kp5078	Case	Urine	Kp	ST36		K102	IS*Kpn74*	1881–1891
Kp5871	Control		Kp	ST14	MDR	K2	IS*Kpn74*	16481–16489
Kp9001	Control		Kp	ST34		K143	IS*Kpn74*	16739–16748; 18390–18398
Kp9063	Control		Kp	ST922		K24	IS*Kpn74*	15789–15797
Kp10193	Case	Urine	Kp	ST3096		K58	IS*Kpn74*	2323–2331
Kp10645	Control		Kv	ST3231		K54	IS*Kpn74*	19038–19046
Kp11667	Case	Blood	Kp	ST15	MDR	K24	IS*Kpn74*	16339–16347
Kp3577	Control		Kp	ST1876		K15	IS*Ec33*	15120–15121
Kp6313	Control		Kp	ST37	MDR	K15	IS*Ec33*	15120–15121
Kp7902	Control		Kp	ST502		K15	IS*Ec33*	15120–15121
Kp8544	Case	Blood	Kp	ST678		K15	IS*Ec33*	15120–15121
Kp8946	Control		Kp	ST1876		K15	IS*Ec33*	15120–15121
Kp3567	Case		Kp	ST2121		K113	IS1*X2*	4776–4784
Kp9457	Control		Kp	ST499		K169	IS1*X2*	26317–26316; 30116–30116
Kp5138	Control		Kp	ST29		K30	IS*Kpn14*	18120–18124
Kp10645	Control		Kv	ST3231		K54	IS*Kpn14*	18369–18374
Kp10769	Case	Blood	Kp	ST11-1LV	MDR	K27	IS*Kpn14*	2159–2167
Kp9457	Control		Kp	ST499		K169	IS*903B*	30177–30185
Kp6973	Control		Kp	ST1401		K49	IS*Ec30*	20604–20612
Kp6973	Control		Kp	ST1401		K49	IS*Sen14*	15478–15479

## DISCUSSION

In this manuscript, we demonstrated that *Kp* strains exhibit capsule loss and, in certain isolates, O-antigen loss, during murine gut colonization (Fig. 1, 3, and 5). This capsule and O-antigen loss impacted fitness at extraintestinal sites as the evolved acapsular isolate Kp4819_M134 had a significant defect in a pneumonia model and in the liver during bacteremia (Fig. 4). The fitness defect was much larger in KPPR1, consistent with prior findings that capsule is critical for lung infection (25) (Fig. S10). The mechanism of capsule loss and acapsular accumulation varied by strain, with high rates and IS-mediated disruption in Kp4819 and low rates through mutation and deletion in KPPR1. IS-mediated capsule mutants were also isolated from patients who were asymptomatically colonized and those who progressed to infection. Although capsule and LPS are considered major virulence factors, these data suggest that changes in these factors across different *Kp* strain backgrounds may support strain adaptability during colonization and alter infection risk over time.

The *Kp* capsule operon has a propensity for undergoing genetic changes, and previous studies have reported capsule inactivation in *Kp* due to various genetic modifications including ISs, deletions, and point mutations (32–34, 53–55). In this study, IS*1X2* was the exclusive cause of capsule mutants in Kp4819, and IS*Kpn26* caused two of three mutations in NJST258_2 (Table 2). The IS*1* family is prone to inserting into AT-rich regions of the genome (56). As the Kp4819 capsule and *rfb* operons have a lower GC content (38% and 39%) than the rest of the genome (57%), this region may have been particularly susceptible to IS*1X2* insertions. In *Klebsiella* isolates from colonized patients, IS*1* transposition from the pOXA-48 plasmid disrupted O-antigen encoding genes including *wbbM,* similar to what we observed in our gut colonization model (42). IS*Kpn26* has also been reported to insert in the capsule operon in an acapsular isolate that evolved in the human urinary tract (57). Accordingly, we found that IS elements disrupted the capsule operon in 7% of highly diverse patient-colonizing strains, in line with previous work where 5.8% of analyzed ST258 isolates had evidence of capsule disruption in the *wbaP*/*wcaJ* gene (34). Combined, this indicates that our gut colonization model recapitulates strain evolution by ISs that occurs in patients.

The observation of *Klebsiella* capsule loss within the host suggests that, under certain conditions, there is a fitness trade-off between capsule presence and absence. Acapsular strains have increased susceptibility to host defense mechanisms such as immune cell phagocytosis and complement-mediated killing (22, 58). In models of infection, acapsular *Klebsiella* strains are significantly attenuated in the lung, highlighting the role of capsule in pathogenesis (25, 59). However, lacking capsules can be beneficial for *Kp* by increasing biofilm formation, cell adhesion, and resistance against polymyxins and phages (60–64). Acapsular strains including *wcaJ* mutants can have increased biofilm formation compared to WT strains (62, 64). In a separate study, isolation of five genetically related *Klebsiella* bloodstream isolates from a single patient over 95 days suggests that IS-mediated inactivation of *wcaJ* may have facilitated *Klebsiella* persistence via decreased capsule production and complement resistance (65). Lack of capsule has also been shown to increase *Klebsiella* adhesion to ileocecal (HCT-8) and bladder (T24) epithelial cells (60, 66). In host sites such as the urinary tract, acapsular strains also have increased persistence and ability to invade bladder epithelial cells (34). Consequently, the increased biofilm formation and adhesion phenotypes of acapsular isolates may allow for *Klebsiella* to better establish and persist in sites such as the gut and urinary tract. Capsule inactivation may also allow strains with susceptible capsule types to escape phage predation, as certain phages have evolved to encode depolymerases for specific capsule serotypes (67, 68). Together, this highlights that capsule loss may facilitate *Kp* establishment and persistence in the host but decrease *Kp* virulence potential at certain sites of infection.

One phenotype associated with capsule loss or inactivation in *Klebsiella* is susceptibility to complement-mediated killing by human serum (22, 51). Although acapsular isolates had a significant decrease in capsule production, *in vivo* isolates were susceptible to killing by human serum, whereas *in vitro* isolates were not (Fig. 2; Fig. S2 and S9). Previous studies have observed variable serum resistance of acapsular *Kp* isolates across strains, complement resistance mechanism, and isolation source (22, 32, 69). Although differences in our strains might be due to the genetic nature of capsule inactivation, O-antigen may also be important as we detected additional ISs that led to changes in O-antigen structure, and mutations outside of capsule and O-antigen operons may also play a role (22, 24, 47, 70). Previous work has defined two potential *Kp* serum resistance mechanisms depending on capsule or LPS (20). In Kp4819, it may be that either capsule or overexpression of O-antigen is sufficient for serum resistance, but that lack of both makes isolates completely susceptible.

The accumulation of acapsular mutants can vary by growth conditions, and there is significantly less inactivation in nutrient-limited compared to rich media (32). Additionally, studies in *Escherichia coli* and *Klebsiella* have demonstrated that low oxygen environments may play a role in oxygen-dependent capsule regulation and biosynthesis (71, 72). In our work, anaerobic conditions inhibited capsule loss *in vitro,* although loss was observed during murine gut colonization (Fig. 1 and 5). As the gut is a nutrient-rich yet dynamic environment with oxygen gradients, these gradients may facilitate capsule loss to varying degrees across the length of the gastrointestinal (GI) tract (73). As capsule loss *in vivo* was lower than during LB passage in our study, the differences in oxygen tensions may contribute to the lower degree of capsule loss observed *in vivo*.

The difference in acapsular mutant accumulation across strains is intriguing and potentially significant. The reasons for these differences are unclear but may include their IS repertoire and differences in their capsule regulons. For example, Kp4819 has IS*1X2* with a propensity for the AT-rich capsule region, whereas NJST258_2 does not, but does have IS*Kpn26* with the ability to disrupt capsule genes. KPPR1, unlike Kp4819 and NJST258_2, is a hypervirulent strain with additional capsule regulators including the *rmpADC* operon, which is regulated by the oxygen-responsive fumarate nitrate reduction regulator (FNR) (71). As IS-mediated gene activation can facilitate rapid strain adaptation, it is important to consider this in the context of patient infection risk. For example, patients colonized with strains with a lower propensity for capsule loss such as KPPR1 may have a higher and prolonged risk of infection, as a greater percentage of the population retains capsule than those colonized with Kp4819 or NJST258_2. A better understanding of the dynamics behind capsule loss across strain backgrounds and host environments could inform patient infection risk.

This work has some limitations. Kp4819, like many *Kp* strains, does not colonize the gut without antibiotic treatment. The ampicillin treatment model used is clinically relevant as many *Kp* patients receive antibiotic treatment. However, we cannot evaluate the impact of gut commensals on capsule loss in an undisrupted community. Additionally, some of the acapsular Kp4819 isolates used for further study had additional mutations outside of the capsule operon. Mutations in the O-antigen operon, for example, may be compensatory for capsule mutations in genes such as *wzy,* which are known to lead to membrane instability (29). Although significant as representative evolved gut isolates, they limit the conclusions we can draw regarding the exact effect of each mutation. Importantly, our complementation experiments were able to show that IS*1* was sufficient to disrupt capsule and serum resistance even in the presence of secondary mutations. In future studies, site-directed, isogenic mutants can be used to separate phenotypes specifically attributable to capsule, O-antigen, and other loci in these isolates.

In conclusion, this study reveals significant strain evolution during gut colonization and suggests that *in vivo* evolution such as capsule and O-antigen loss may have implications in clinical settings. As the capsule is important for virulence at extraintestinal sites, patients colonized with *Kp* strains with a propensity for capsule loss may have a lower risk of infection than a patient colonized with a strain that does not readily undergo capsule loss. Further work is needed to explore mechanisms of strain evolution during colonization across additional *Kp* strains and the impact of this genetic evolution on patient infection risk.

## MATERIALS AND METHODS

### Bacterial strains and media

Kp4819 was isolated from the rectal swab of a patient enrolled in a previous case-control study (43). All *Kp* strains were cultured overnight in LB broth (Fisher Scientific, Waltham, MA) at 37°C shaking or grown on LB agar (Fisher Scientific, Waltham, WA) plates at 27°C. The bacterial strains used in this work are described in Table S1. For details regarding the construction of mutants and marked strains, see the supplemental methods.

### DNA extraction and sequencing

Acapsular isolates were grown overnight, and DNA was extracted using the DNeasy UltraClean Microbial Kit (Qiagen, Hilden, Germany). Samples were submitted to the University of Michigan Advanced Genomics Core for Illumina sequencing. DNA from the wild-type Kp4819 and KPPR1 and NJST258_2 isolates was extracted using the Wizard Genomic DNA Purification Kit (Promega, Madison, WI) and submitted to plasmidsaurus (Eugene, OR) for Illumina and ONT sequencing. Primers flanking the locations of potential ISs were used to generate amplicons; amplicons were purified using the QIAquick PCR Purification Kit (Qiagen, Hilden, Germany) and submitted to Azenta Life Sciences (Burlington, VT) for Sanger sequencing (Table S2).

### Bioinformatic analysis

Assemblies were generated using the Nanosake workflow (https://github.com/Snitkin-Lab-Umich/Nanosake). Variant calling was performed with a customized variant calling pipeline (https://github.com/Snitkin-Lab-Umich/snpkit) with hybrid assemblies of the respective WT strains used for variant calling. Insertion sequences were identified using panISa 0.1.7 and a customized variant calling pipeline (https://github.com/kylegontjes/ISScreener) (46, 52). See supplemental methods for details.

### Murine models

Murine models were performed using 8- to 12-week-old C57Bl/6 mice (Jackson Laboratory, Bar Harbor, ME). For competitions, strains were mixed 1:1 and plated on LB agar with or without kanamycin (40 µg/mL). To model pneumonia, mice were infected retropharyngeally with either 1 × 10^8^ CFU Kp4819 and derivative mutants or 1 × 10^6^ CFU of KPPR1 and derivative mutants. For models of bacteremia, mice received a tail vein injection of 1 × 10^7^ CFU of *Kp*. For gut colonization models, mice received 0.5 g/L of ampicillin via their drinking water for 4 days prior to inoculation. Mice were inoculated with 5 × 10^6^ CFU of *Kp* via oral gavage and remained on ampicillin for the duration of the experiment. Fecal pellets were collected, homogenized in phosphate-buffered saline (PBS), and plated on LB agar with ampicillin (36 µg/mL) or rifampin (30 µg/mL).

### Growth assays and LB passaging

To assess *in vitro* growth and replication, *Kp* strains were grown overnight and adjusted to 1 × 10^7^ CFU/mL in LB or M9+glucose. The OD_600_ was measured using an Eon microplate reader and Gen5 software (BioTek, Winooski, VT) every 15 minutes for 12 hours.

For LB passaging, 5 µL of the inoculum was added to 5 mL of LB for a final concentration of 5 × 10^5^ CFU/mL. Every 24 hours, 100 µL from each tube of LB was serially diluted and plated. Cultures were diluted 1:1,000 in fresh media every 24 hours. For comparisons of aerobic and anaerobic conditions, a single bacterial colony was resuspended in PBS and added to 5 mL of LB in a culture tube. A single colony was used to avoid the development of pale colonies during overnight growth. Culture tubes were incubated aerobically or in an anaerobic jar with a BD GasPak (BD, Franklin Lakes, NJ) at 37°C and serially passaged as stated above. LB for anaerobic passaging was pre-reduced for 24 hours before inoculation.

### Serum killing assay

Serum killing assays were performed using pooled human serum from Innovative Research Inc., Novi, MI, or deidentified, to-be-discarded serum from the University of Michigan Medical Center as described previously (74). Killing was measured via serial dilutions and plating at time 0 and after 3 hours.

### Uronic acid quantification assays

Extracellular uronic acids were quantified as described previously (75, 76). The background was subtracted from each sample; uronic acid levels were quantified based on a standard curve and normalized to the starting OD_600_. See the supplemental methods for additional details.

### LPS extraction and visualization

*Klebsiella* strains were grown overnight, and then LPS was isolated from 1 × 10^9^ CFU of *Kp* strains of interest using the Sigma Lipopolysaccharide isolation kit (Sigma Aldrich, St. Louis, MO) as described previously (49). Then 10 µL of LPS from each strain was run on a 4%–20% mini-PROTEAN TGX gel (Bio-Rad, Hercules, CA) and stained using the Pro-Q Emerald 300 Stain Kit (Molecular Probes, Eugene, OR).

### Statistical analysis

All *in vivo* experiments were performed independently at least two times, and all *in vitro* experiments were performed with a minimum of three independent biological replicates unless otherwise stated. All data were analyzed and visualized using GraphPad (GraphPad Software, La Jolla, CA). A Dunnett’s multiple comparisons test was used for data in which comparisons were made to a wild-type control, while the Šídák’s multiple comparisons test was used for experiments in which selected comparisons were made across multiple independent samples. Significance was defined as a *P*-value ≤0.05.

## Data Availability

All sequencing reads are available from the NCBI SRA database (PRJNA1174114 and PRJNA789565).

## SUPPLEMENTAL MATERIAL

Supplemental Material mbio.02362-24-s0001.pdf Supplemental figures, tables, and methods.

Table S3 mbio.02362-24-s0002.xlsx Genome information of human rectal swab isolates used in this study.

ASM does not own the copyrights to Supplemental Material that may be linked to, or accessed through, an article. The authors have granted ASM a non-exclusive, world-wide license to publish the Supplemental Material files. Please contact the corresponding author directly for reuse.
