# Supplementary material for: Klebsiella pneumoniae evolution in the gut leads to spontaneous capsule loss and decreased virulence potential
Source: mBio. 2025 Mar 31;16(5):e02362-24. doi: 10.1128/mbio.02362-24 (PMC12077207; doi:10.1128/mbio.02362-24)
Supplement: Supplemental Material — Supplemental figures, tables, and methods. [file mbio.02362-24-s0001.pdf]

Supplemental Information

Supplemental Figures

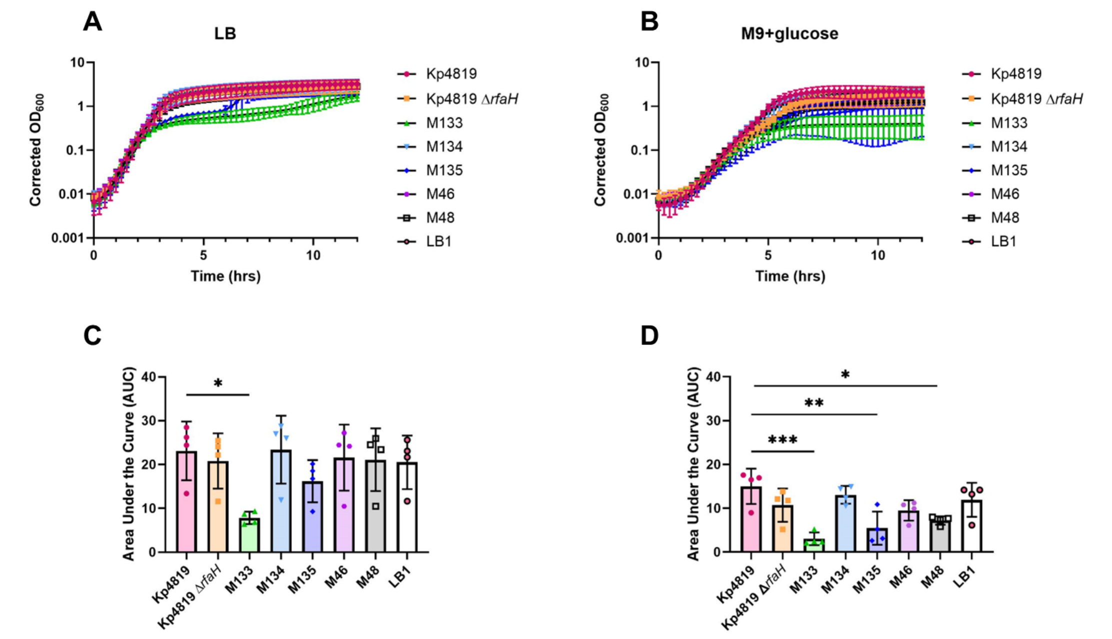

Supplemental Figure 1: Majority of Acapsular Kp4819 Isolates Do Not Display Replication Defects During *in vitro* Growth

The *in vitro* replication of Kp4819, Kp4819 Δ*rfaH*, and six acapsular Kp4819 isolates was measured in LB (A,C) and M9+glucose (B,D). The OD_{600} (mean with SD) is displayed in (A) and (B) and quantified as area under the curve in C and D. For A-D, *, p< 0.05; **, p<0.01; ***, p<0.001, by one-way ANOVA with an n=4.

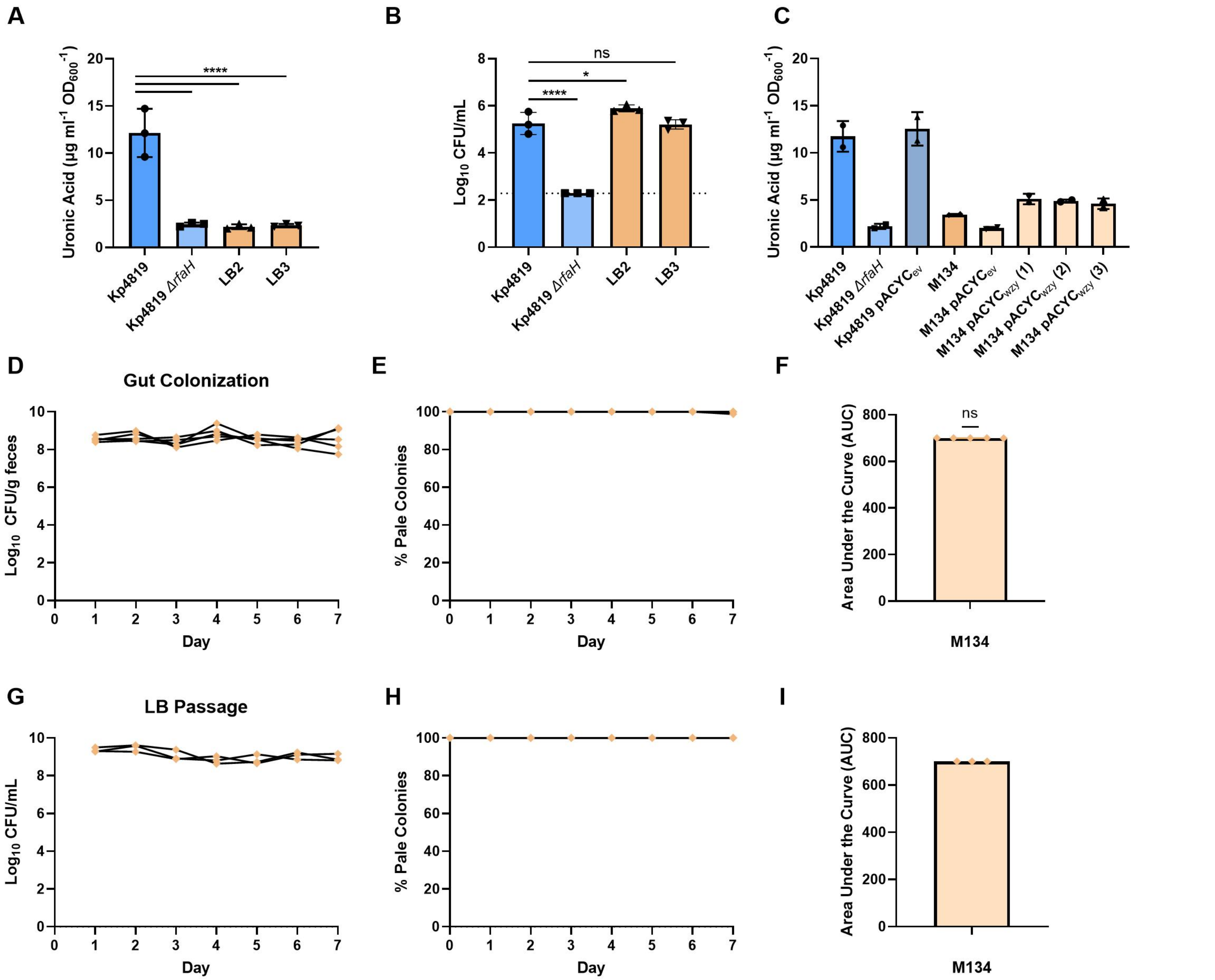

Supplemental Figure 2: Characterization of Acapsular Kp4819 Isolates *in vitro* and *in vivo*

Acapsular Kp4819 Isolates from LB passaging and mouse gut colonization experiments were characterized *in vitro* and *in vivo*. Capsule production of Kp4819, Kp4819 Δ*rfaH*, and two additional Kp4819 pale isolates from LB passaging was determined via uronic acid quantification (A). Susceptibility to human serum after three hours was compared for $1x10^5$ CFU of Kp4819, Kp4819 Δ*rfaH*, and two additional LB passaging Kp4819 isolates (LB2-3) after three hours (B). Capsule production of Kp4819, Kp4819 Δ*rfaH*, and single gene complemented Kp4819_M134 clones (M134_pACYC$_{wzy}$ 1-3) was measured via uronic acid quantification (C). The Kp4819_M134 acapsular

isolate was used to inoculate mice with $5x10^6$ CFU in a murine gut colonization model (D-F) and 5 μL of the inoculum was passaged concurrently in 5 mL of LB (G-I). *Kp* levels during gut colonization or LB passage were measured by plating and displayed as $\log_{10}$ CFU/g feces (D) or $\log_{10}$ CFU/mL (G). The percentage of pale colonies was monitored for during gut colonization and LB passage and displayed as percentages (E,H) and as area under the curve (F,I) with mean and SEM. For (A and B), by one-way ANOVA compared to the Kp4819 WT with n=3 with a Dunnett's multiple comparisons test and *, p<0.05; **, p<0.01; ***, p<0.001; ****, p<0.0001. For (C), n=2, no statistics were performed. For (D-F), n=5 and for (G-I), n=3. For (F), there was no significant difference by one sample t-test with a hypothetical mean = 700 (the area of 100% pale colonies over 7 days). For I, the standard deviation was 0 as all values were equal to 700, so no statistics could be performed.

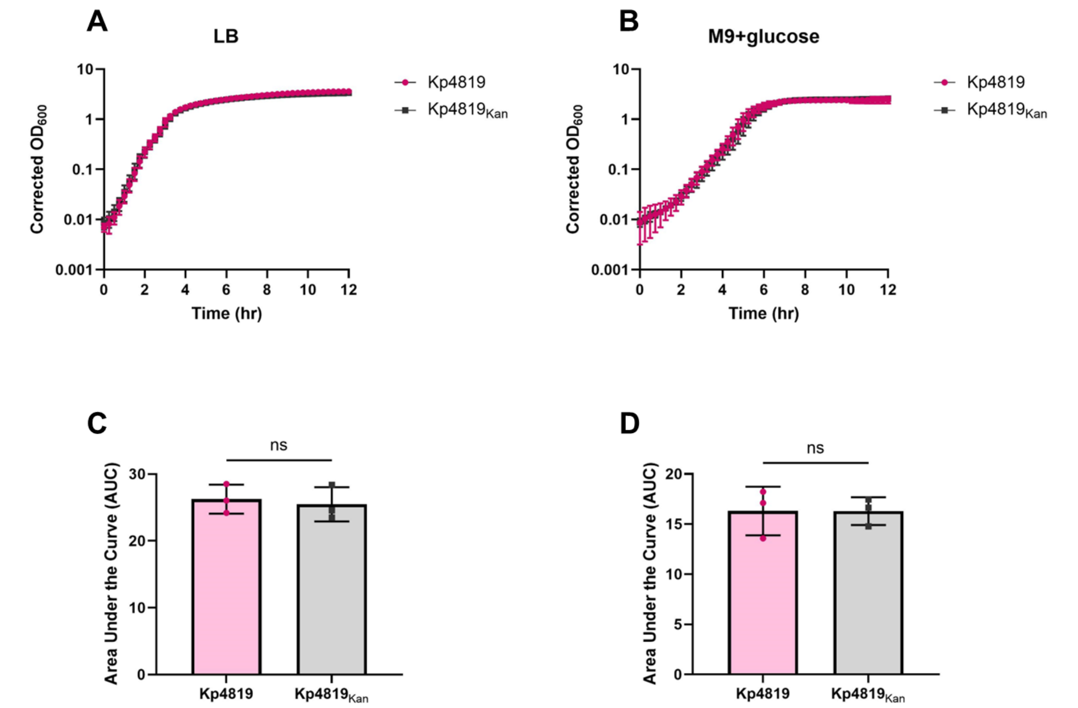

Supplemental Figure 3: $Kp4819_{Kan}$ Has Equivalent *in vitro* Growth Kinetics as Kp4819

The *in vitro* replication of Kp4819 and $Kp4819_{Kan}$ was measured in LB (A,C) and M9+glucose (B,D) over 12 hours. The OD_{600} (mean with SD) is displayed in (A) and (B) and quantified as area under the curve in (C) and (D). For (C-D), n=3 with no significant difference with a paired t-test.

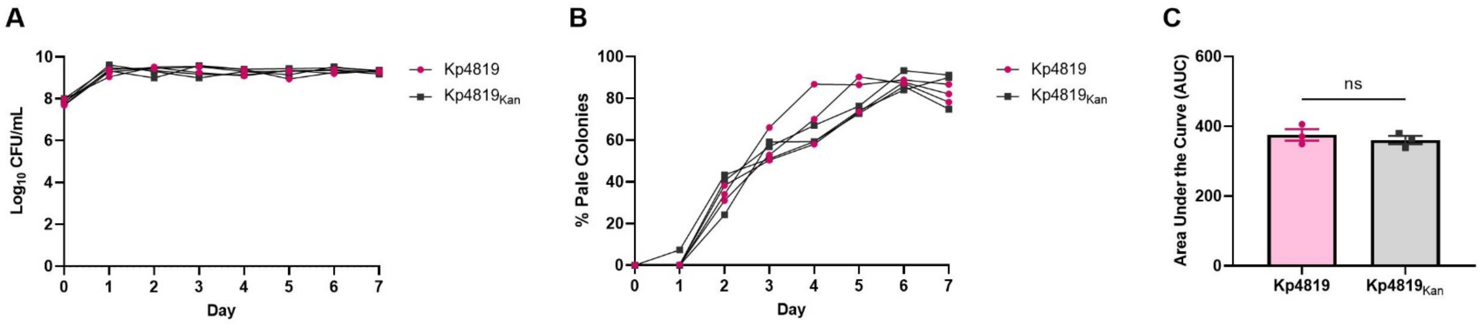

Supplemental Figure 4: Kp4819$_{Kan}$ Has the Same Propensity for *in vitro* Capsule Loss as Kp4819

The Kp4819 WT and Kp4819$_{Kan}$ were passaged in 5 mL of LB for 7 days and LB cultures were diluted 1:1000 into 5 mL of fresh LB broth every 24 hours. The total bacteria (A) and percent pale colonies (B) were assessed via plating. The percent pale colonies were quantified as area under the curve (C). For (C), n=3 with no significant difference based on a paired t-test.

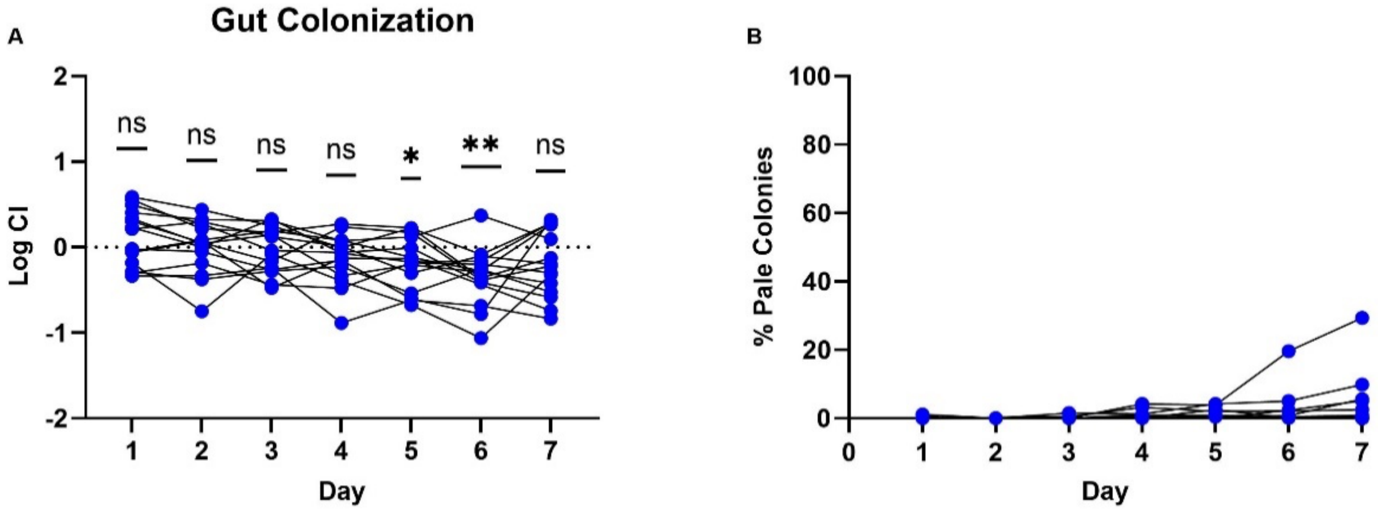

Supplemental Figure 5: Kp4819_M134 Does Not Have a Fitness Defect During Gut Colonization

Kp4819$_{Kan}$ was competed 1:1 against Kp4819_M134 in a murine gut colonization model. Mice were inoculated with $5x10^6$ CFU of *Kp* via oral gavage. The mean log_{10} competitive index (A) and percent pale colonies of Kp4819$_{Kan}$ (B) are displayed. For (A), *, p< 0.05; **, p<0.01; ****, p<0.0001, by one sample t-test and n=15 performed across three independent experiments.

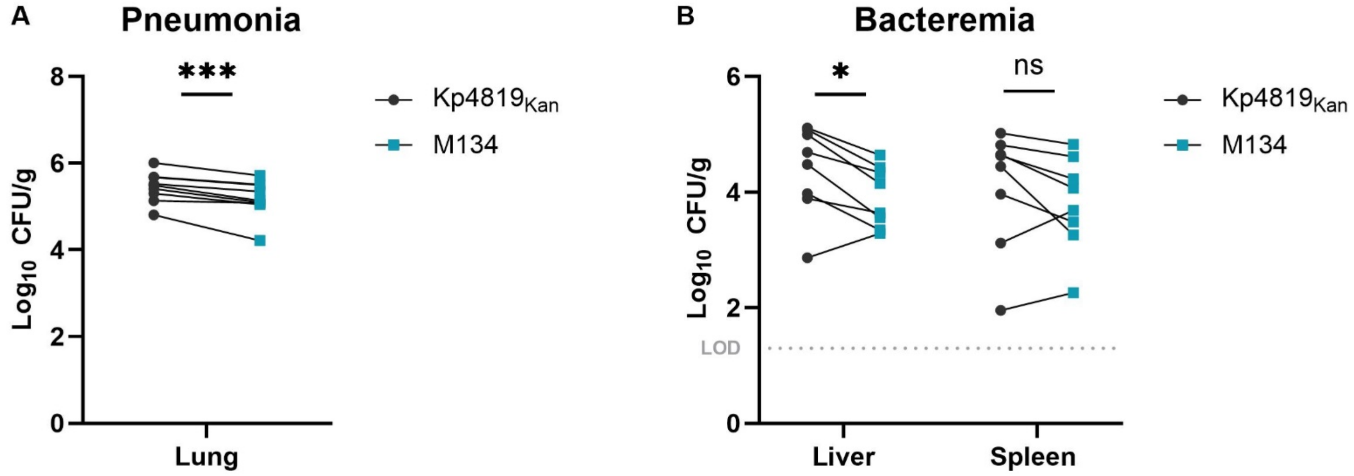

Supplemental Figure 6: Bacterial Burden During Models of Extraintestinal Infection

The bacterial burden of $Kp4819_{Kan}$ and Kp4819_M134 in a murine pneumonia (A) or bacteremia (B) model after 24 hours is quantified on a log_{10} scale. For (A) n=10, and for (B) n=8, using a paired t-test with *, $p<0.05$; ***, $p<0.001$.

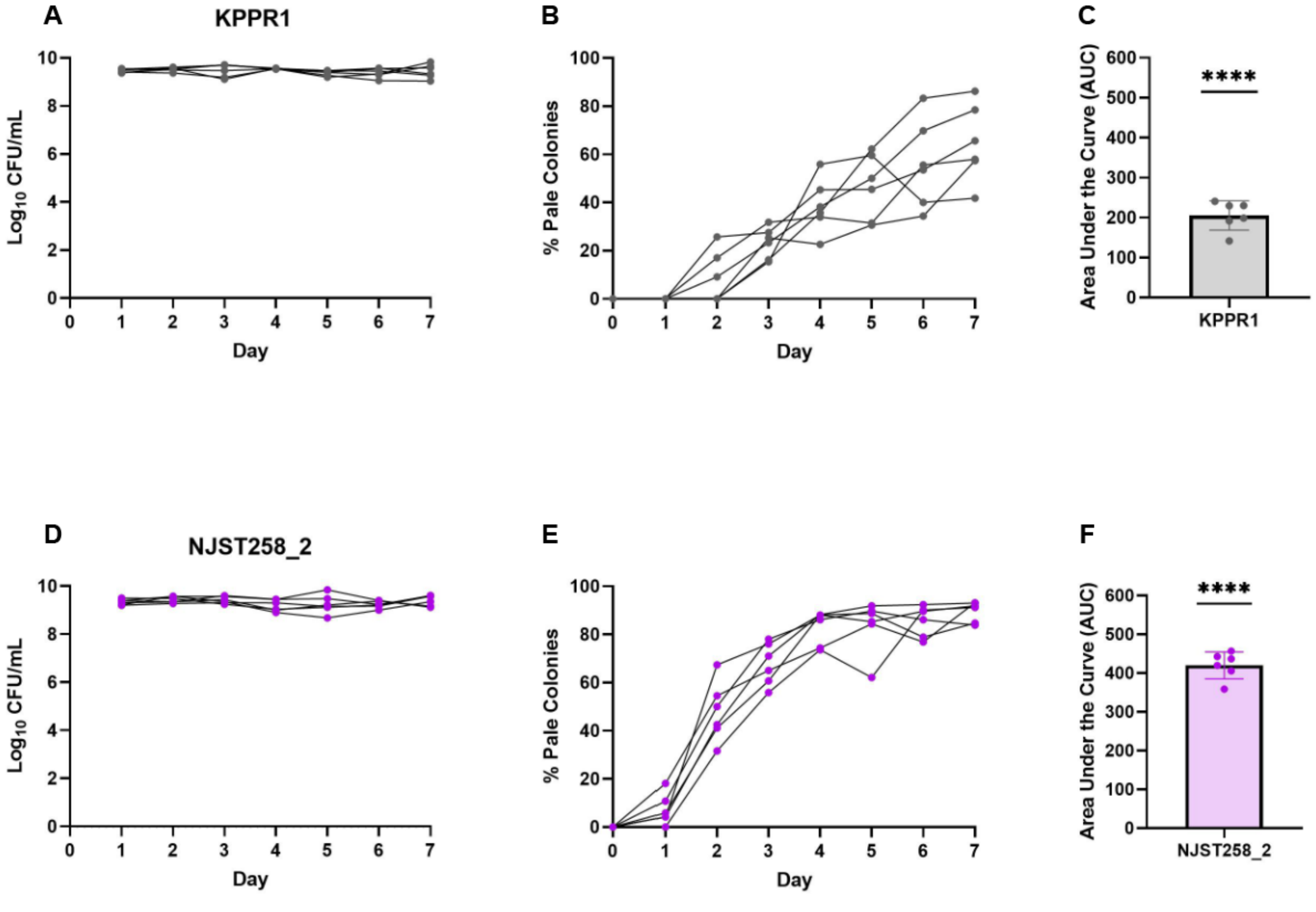

Supplemental Figure 7: KPPR1 and NJST258_2 Lose Capsule to Significant Degrees During LB Passage

The inoculum used for each of the KPPR1 and NJST258_2 murine gut colonization experiments were used to perform concurrent LB passaging experiments. For each strain 5 µL of the inoculum was added to 5 mL of LB and passaged concurrently with the murine gut colonization experiment. Cultures were diluted 1:1000 into 5 mL of fresh LB every 24 hours. The total amount of bacteria (A, D) and development of pale colonies was monitored for during LB passage and displayed as percentages (B,E) and as area under the curve (C, F) with mean and SEM. For (C) and (F), ****, $p<0.0001$ by one sample t-test with a hypothetical mean = 0. For (A-F), n=6 across 2 independent experiments.

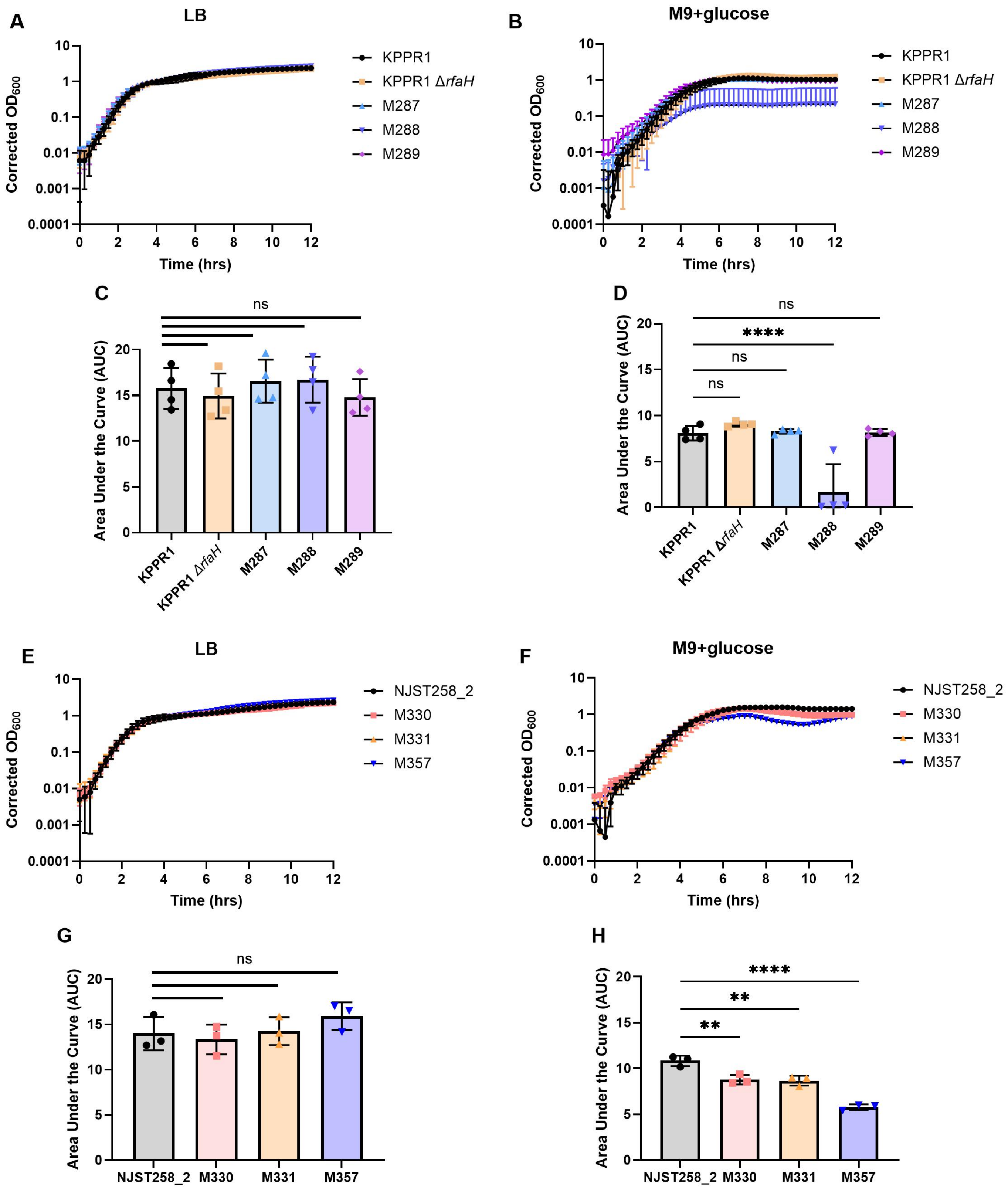

Supplemental Figure 8: KPPR1 and NJST258_2 Display Replication Defects During *in vitro* Growth in M9+glucose but Not LB

The *in vitro* replication of KPPR1, KPPR1 Δ*rfaH*, and three acapsular KPPR1 isolates was measured in LB (A,C) and M9+glucose (B,D). The *in vitro* replication for the NJST258_2 WT and three acapsular isolates was measured in LB (E,G) and M9+glucose (F,H). The OD600 (mean with SD) is displayed in (A,B,E,F) and quantified as area under the curve in (C,D,G,H). For (C,D,G,H), **, $p<0.01$; ***, $p<0.001$; ****, $P<0.0001$, by one-way ANOVA with an $n>=3$.

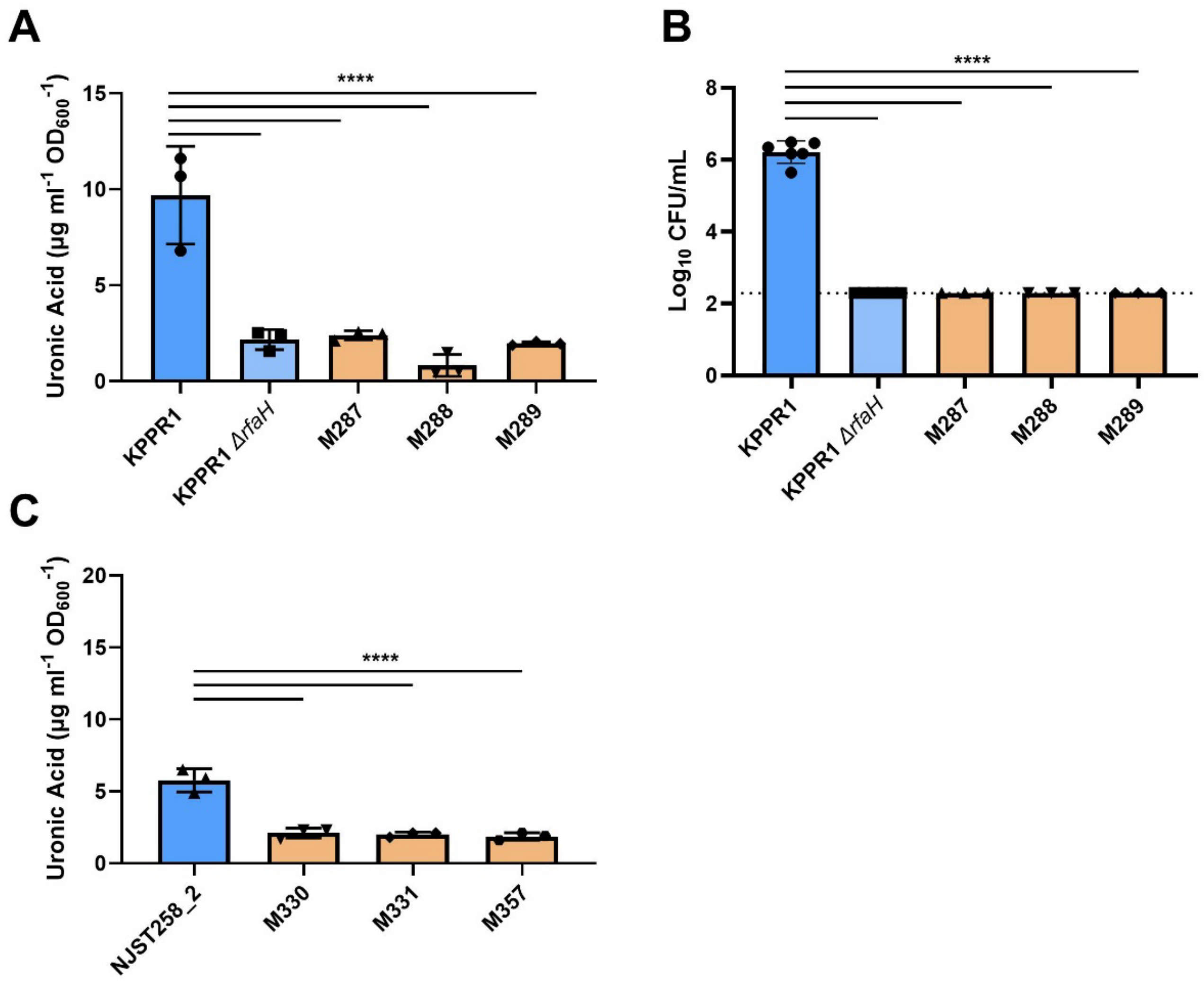

Supplemental Figure 9: KPPR1 and NJST258_2 Pale Isolates from the Gut Are Acapsular

Capsule production of KPPR1 (A) and NJST258_2 (C) pale strains was determined via uronic acid quantification. Human serum susceptibility after three hours of 1×10^5 CFU of KPPR1, the acapsular KPPR1 Δ*rfaH* mutant, and three acapsular KPPR1 isolates (KPPR1_M287-289) from mouse gut colonization experiments was determined (B). For (A-C), statistical significance between the KPPR1 WT or NJST258_2 WT and each mutant was determined by one-way ANOVA with $n \geq 3$, with a Dunnett's multiple comparisons test with ****, $p<0.0001$.

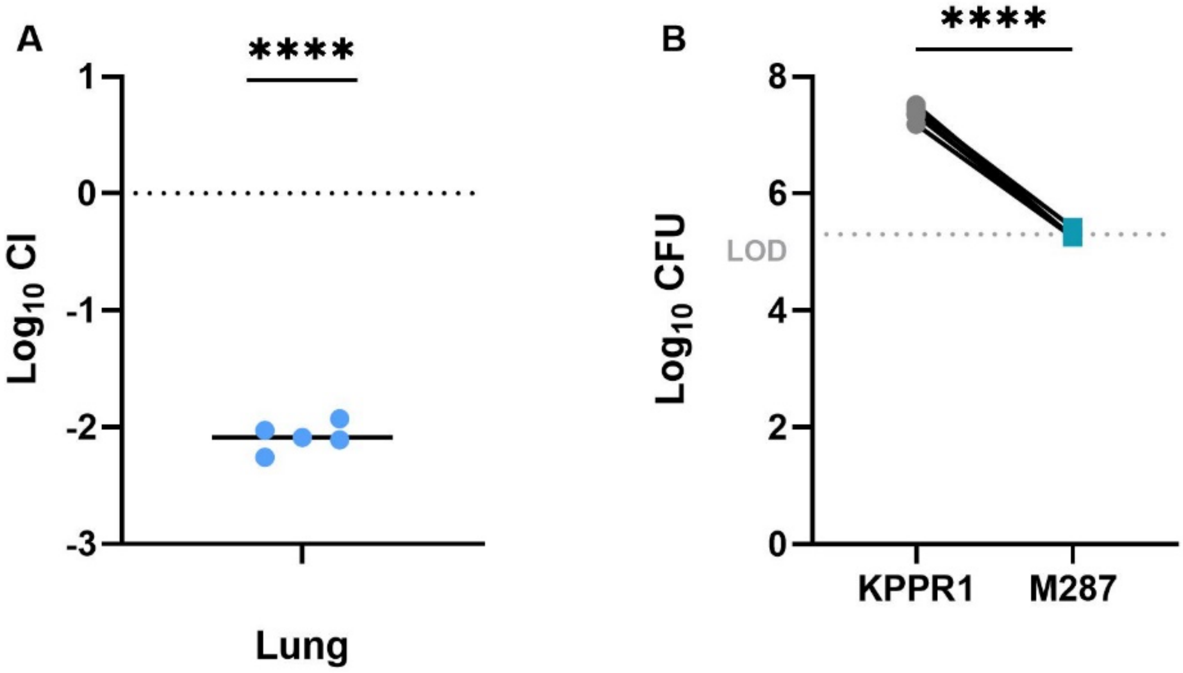

Supplemental Figure 10: An Evolved Acapsular KPPR1 Isolate Has a Significant Fitness Defect in the Lung

KPPR1 marked with kanamycin and KPPR1_M287 were competed 1:1 in a murine pneumonia model. Mice were inoculated with $1x10^6$ CFU of a 1:1 mixture of KPPR1 marked with kanamycin (KPPR1 STAMPR) and KPPR1_M287 retropharyngeally. The log_{10} competitive index (A) and bacterial burden (B) in the lung after 24 hours are displayed. For (A), n=5 with a one sample-test and ****, p<0.0001. For (B), n=5, with ****, p<0.0001 determined by paired t-test.

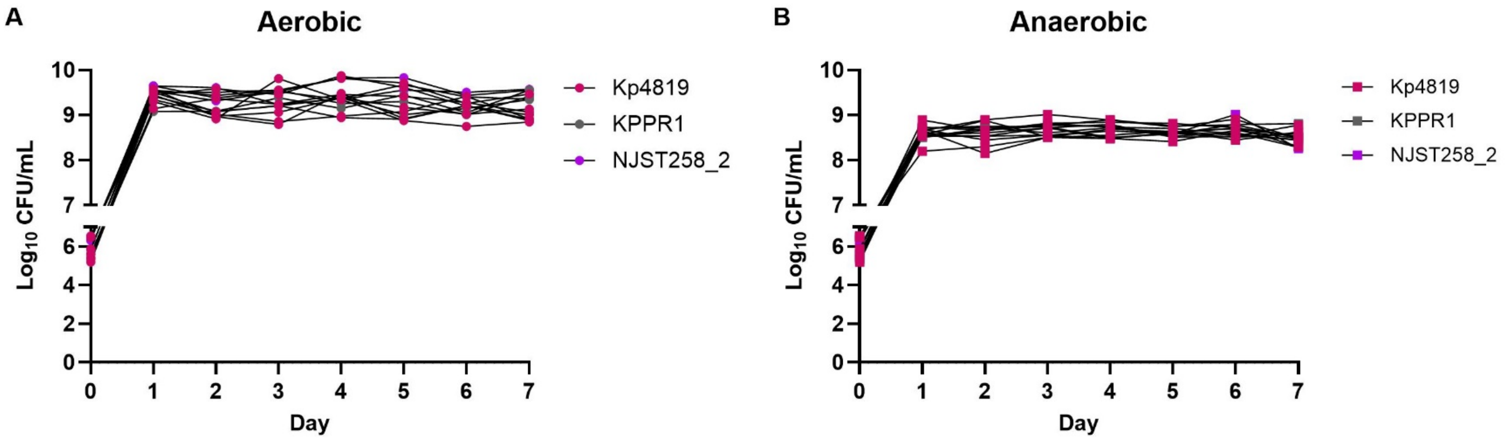

Supplemental Figure 11: Total Bacterial CFU/mL of *Kp* Strains During Aerobic and Anaerobic Passage

The total bacterial CFU/mL of Kp4819, KPPR1, and NJST258_2 during passage in 5 mL of LB under aerobic (A) or anaerobic (B) conditions is quantified on a $\log_{10}$ scale. Every 24 hours, LB cultures were diluted 1:1000 into 5 mL of fresh LB broth. For Kp4819, n=8, for KPPR1 and NJST258_2 n=3.

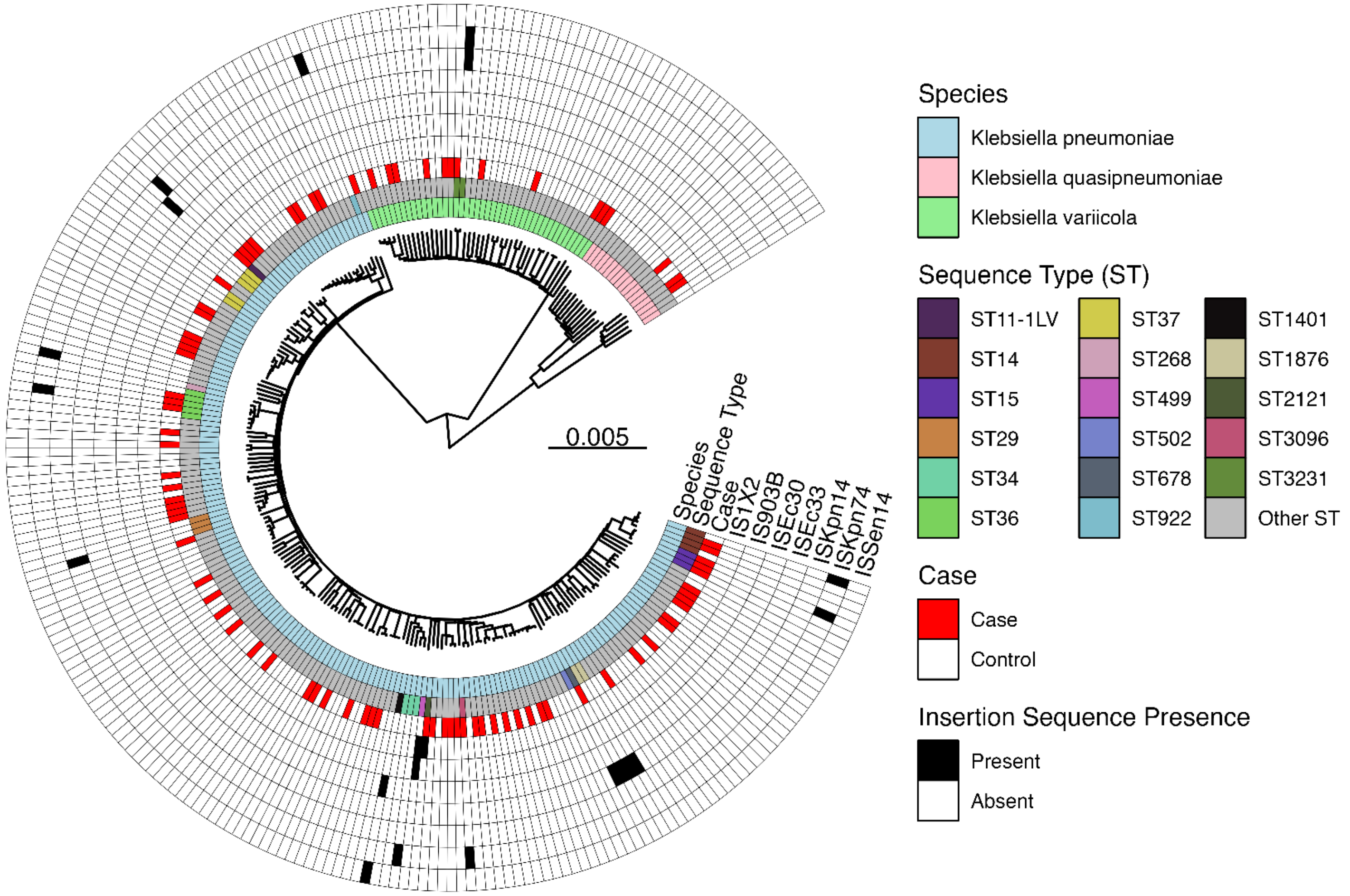

Supplemental Figure 12: Phylogenetic Tree of *Klebsiella* Rectal Isolates

A phylogenetic tree of *Klebsiella* rectal isolates annotated with species, case status, and the presence of insertion sequences in the capsule operon. The phylogenetic tree was constructed using the neighbor-joining method, with an amino acid distance matrix generated from the single-copy core gene alignment by the R package, cognac. Only sequence types that have strains with insertion sequences in the capsule operon were displayed.

Supplemental Table 1. List of Bacterial Strains Used in this Study.

Species	Strain	Description	Reference (PMID)
Klebsiella pneumoniae	Kp4819	Rectal isolate from a colonzied patient	35915063
Klebsiella pneumoniae	Kp4819 Δ*rfaH*	Kp4819 with a kanamycin resistance gene in place of *rfaH*	
Klebsiella pneumoniae	Kp4819 pkD46	Kp4819 containing the pkD46 plasmid	
Klebsiella pneumoniae	Kp4819$_{Kan}$	Kp4819 with a kanamycin resistance gene in an intergenic region	
Klebsiella pneumoniae	Kp4819 pACYC$_{ev}$	Kp4819 with the pACYC184 vector	
Klebsiella pneumoniae	Kp4819_M133	Kp4819 acapsular strain isolated from feces of a mouse colonized with Kp4819 in the gut	
Klebsiella pneumoniae	Kp4819_M134	Kp4819 acapsular strain isolated from feces of a mouse colonized with Kp4819 in the gut	
Klebsiella pneumoniae	Kp4819_M135	Kp4819 acapsular strain isolated from feces of a mouse colonized with Kp4819 in the gut	
Klebsiella pneumoniae	Kp4819_M46	Kp4819 acapsular strain isolated from feces of a mouse colonized with Kp4819 in the gut	
Klebsiella pneumoniae	Kp4819_M48	Kp4819 acapsular strain isolated from feces of a mouse colonized with Kp4819 in the gut	
Klebsiella pneumoniae	Kp4819_LB1	Kp4819 acapsular strain isolated from *in vitro* passaging in LB	
Klebsiella pneumoniae	Kp4819_LB2	Kp4819 acapsular strain isolated from *in vitro* passaging in LB	
Klebsiella pneumoniae	Kp4819_LB3	Kp4819 acapsular strain isolated from *in vitro* passaging in LB	
Klebsiella pneumoniae	Kp4819_M134 pkD46	M134 containing the pkD46 plasmid	
Klebsiella pneumoniae	Kp4819_M134 pACYC$_{ev}$	M134 with the pACYC184 vector	
Klebsiella pneumoniae	Kp4819_M134 pACYC$_{wzy}$ (1)	M134 with the pACYC vector containing the wzy gene	
Klebsiella pneumoniae	Kp4819_M134 pACYC$_{wzy}$ (2)	M134 with the pACYC vector containing the wzy gene	
Klebsiella pneumoniae	Kp4819_M134 pACYC$_{wzy}$ (3)	M134 with the pACYC vector containing the wzy gene	
Klebsiella pneumoniae	Kp4819_M134 ΔIS*1* (1)	M134 with the IS removed from *wzy*	
Klebsiella pneumoniae	Kp4819_M134 ΔIS*1* (2)	M134 with the IS removed from *wzy*	
Klebsiella pneumoniae	Kp4819_M134 ΔIS*1* (3)	M134 with the IS removed from *wzy*	
Klebsiella pneumoniae	KPPR1		25291761
Klebsiella pneumoniae	KPPR1 STAMPR	KPPR1 marked with kanamycin in the Tn7 site	
Klebsiella pneumoniae	KPPR1 Δ*rfaH*	KPPR1 with a kanamycin resistance gene in place of *rfaH*	
Klebsiella pneumoniae	KPPR1_M287	KPPR1 acapsular strain isolated from feces of a mouse colonized with KPPR1 in the gut	
Klebsiella pneumoniae	KPPR1_M288	KPPR1 acapsular strain isolated from feces of a mouse colonized with KPPR1 in the gut	
Klebsiella pneumoniae	KPPR1_M289	KPPR1 acapsular strain isolated from feces of a mouse colonized with KPPR1 in the gut	
Klebsiella pneumoniae	NJST258_2		24639510
Klebsiella pneumoniae	NJST258_2_M330	NJST258_2 acapsular strain isolated from feces of a mouse colonized with NJST258_2 in the gut	
Klebsiella pneumoniae	NJST258_2_M331	NJST258_2 acapsular strain isolated from feces of a mouse colonized with NJST258_2 in the gut	
Klebsiella pneumoniae	NJST258_2_M357	NJST258_2 acapsular strain isolated from feces of a mouse colonized with NJST258_2 in the gut	
	**Plasmids**		
	pkD46	Plasmid containg lambda phage recombination machinery	10829079
	pkD4	Vector with kanamycin resistance cassette	10829079
	pACYC184	Cloning vector with chloramphenicol resistance	
	pACYC$_{wzy}$	Cloning vector containing the *wzy* gene from the Kp4819 WT	

Supplemental Table 2. List of Primers Used in This Study.

Primer Name	Sequence	Purpose	Reference
rfaH λ Red F	TTTCGCGATTACACCAGCCATTTGTTATGCTTGCCGTTATTAGAAGTGGAATGAGTCATTGTGTAGGCTGGAGCTGCTTC	Lambda Red Primer for rfaH	26060277
rfaH λ Red R	GCTGGAGGCGCAAACGGACGCAAACGTCAGGGCGCTGTTTGGCGTCGACATTAACGGCGTATGGGAATTAGCCATGGTCC	Lambda Red Primer for rfaH	26060277
111	AGATTGGCGCCAGCCGCGTGGTGATGACCATGGTTGGCGGAAACGTCGTCTTTCAAGACTAACCGGTGTAGGCTGGAGCTGCTTC	Insert a Kanamycin resistance cassette into a chromosomal intergenic region in Kp4819	
112	CATTTTCGAGTTATCCCTGCGAAATCTGCTTTAATTCGCGCCTGTAGCGGAAGAACGTTGCCCCTATGGGAATTAGCCATGGTCC	Insert a Kanamycin resistance cassette into a chromosomal intergenic region in Kp4819	
151	CGTGGTATCACTAACAATAC	Amplify the IS sequence in the capsule operon of	
152	CATCCATGCCATATCTAGT	Amplify the IS sequence in the capsule operon of M133 and LB1	
153	CCTTGGTTAGGATTTGTAGT	Amplify the IS sequence in the capsule operon of	
154	GACGGAGCACAGAATTACA	Amplify the IS sequence in the capsule operon of	
155	GATGCTAATGCTCTATCAAGC	Amplify the IS sequence in the capsule operon of	
156	GTGTATTTCATACCGAGTGG	Amplify the IS sequence in the capsule operon of	
157	GTCAGCACTTGACGATAAG	Amplify the IS sequence in the capsule operon of	
158	TGGTGTGTTTACTGCTGT	Amplify the IS sequence in the capsule operon of	
159	TACTCCACCAATTATAGGCA	Amplify the IS sequence in the capsule operon of	
162	CTGCAAATGGTGTAGATCA	Amplify the IS sequence in the capsule operon of	
163	CAGCATCACGTCCATATAC	Amplify the IS sequence in the capsule operon of	
178	TCGTATCGCATAATGCAGC	Primer for IS sequence removal in M134	
179	AGCATCGTAGTCTTGCCAAGC	Primer for IS sequence removal in M134	
186	GCAGCTTCTGGGATG	Confirmation primers for the IS in the capsule operon of Kp7772	
187	GCTTCCACGTACCAG	Confirmation primers for the IS in the capsule operon of Kp7772	
190	CAGCGACTACGATCTGG	Confirmation primers for the IS in the capsule operon of Kp3567 and Kp6146	
191	CAGTCTGCATCTGGTGC	Confirmation primers for the IS in the capsule operon of Kp3567 and Kp6146	
192	TTGGCATCCCAGAGC	Confirmation primers for the IS in the capsule operon of Kp5138 and Kp6320	
193	GTGCTACAATAACACCTGT	Confirmation primers for the IS in the capsule operon of Kp5138 and Kp6320	

Supplemental Table 3. Genome and Kleborate data for human rectal swab isolates used in this study. See separate file. Abbreviations are as follows: *K. pneumoniae* (Kp), *K. variicola* (Kv), and *K. quasipneumoniae* (Kqp), Sequence Type (ST).

Supplemental Methods

Lambda Red Mutagenesis

Kp4819 strains marked with antibiotic resistance cassettes were constructed using Lambda Red mutagenesis as described previously[1]. Briefly, a kanamycin cassette was amplified from the pkD4 plasmid using primers also containing 65 bp homologous to regions flanking the gene to be disrupted. Then, Kp4819 transformed with the pkD46 plasmid encoding the Lambda red machinery was grown in the presence of L-arabinose to induce the expression of the lambda red recombinase. After induction, the amplicons containing the kanamycin resistance cassette and homologous regions were electroporated into Kp4819 pkD46 and samples were recovered overnight at 27°C. Successful transformants were selected by plating on LB agar plates containing kanamycin at 37°C and successful mutants or marked strains were confirmed by PCR. To remove the IS disrupting the Kp4819_M134 capsule operon, a 1376 bp region encoding the Kp4819 WT *wzy* gene was amplified from Kp4819. Then Kp4819_M134 was transformed with the pkD46 plasmid and the same lambda red induction process was used as stated above to induce lambda red recombinase expression. The 1376 bp amplicon was then electroporated into the induced Kp4819_M134 pkD46 strain and colonies were recovered overnight at 27°C. To select for successful transformants with a wild-type copy of the *wzy* gene and restored capsule production, recovered cells were then mixed with human serum at a 1:10 ratio and incubated at 37°C for 3 hours. Incubated serum samples were plated on LB plates and incubated overnight at 37°C. Candidate colonies were then screened by colony PCR to confirm IS removal based on amplicon size.

Uronic acid quantification assays

The OD_{600} of overnight *Kp* cultures was measured. Cultures were spun down, resuspended in 1mL of PBS, and combined with 0.2 mL of Zwittergent in citric acid and incubated for 20 min at 50°C. Bacteria were pelleted and 250 µL of the supernatant was mixed with 1 mL of 80% ethanol and

incubated for 20 min at 4°C. Bacteria were pelleted and resuspended in 166.7 µL of water. Then 1 mL of sodium tetraborate in sulfuric acid was added and samples were incubated for 5 min at 100°C. Then 16.7 µL 3-hydroxydiphenyl solution was added and the OD_{600} was measured. A control sample with 16.7 µL of sodium hydroxide was used to measure background OD_{600}.

Bioinformatic Analysis

Hybrid Assembly

Long read and Illumina reads were used to generate hybrid assemblies using the Nanosake workflow (https://github.com/Snitkin-Lab-Umich/Nanosake). Filtlong was used to remove low quality reads and reads less than 1000 bp (--min_length 1000 --keep_percent 95). Clean filtlong nanopore reads were assembled with Flye assembler and polished with long reads using Medaka, followed by polishing with clean trimmed Illumina reads using Polypolish - to generate Flye+Medaka+Polypolish assembly. For the short Illumina read first approach, long reads were assembled in hybrid mode using Unicycler - medium mode, followed by polishing with short reads using Polypolish. Prokka was used for assembly annotation, BUSCO for assembly completeness statistics and QUAST for assembly statistics.

SNV/Indel Analysis using SNPKIT

Variant calling on assembled genomes was performed with a customized variant calling pipeline (https://github.com/Snitkin-Lab-Umich/snpkit). The quality of sequencing reads was assessed with FastQC v0.11.9, and Trimmomatic v0.39 was used for trimming adapter sequences and low-quality bases. Soft and hard clipped alignments were filtered using samclip v0.4.0. Single nucleotide variants (SNVs) were identified by mapping filtered reads to the hybrid Kp4819 assembly using the Burrows-Wheeler short-read aligner (bwa v0.7.17), discarding polymerase chain reaction duplicates with Picard v3.0.0, and calling variants with SAMtools and bcftools v1.9. Variants were filtered from raw results using VariantFiltration from GATK v4.5.0.0 (QUAL >100; MQ >50; >=10 reads supporting

variant; and FQ < 0.025). Indels were called using the GATK HaplotypeCaller with the following filters: root mean square quality (MQ) > 50.0, GATK QualbyDepth (QD) > 2.0, read depth (DP) > 9.0, and allele frequency (AF) > 0.9. In addition, a custom Python script was used to filter out (mask) variants in the whole-genome alignment that were: SNVs <5 base pairs (bp) in proximity to indels, in a recombinant region identified by Gubbins v3.0.0, in a phage region identified by the PHASTEST web tool or they resided in tandem repeats of length greater than 20bp as determined using the exact-tandem program in MUMmer v3.23[2]. SnpEff was used to predict the functional impact of single nucleotide variants and indels (high, moderate, low, modifier)

Insertion Sequence Identification Using panISa

The tool panISa was used to identify insertion sequences *ab initio* from short-read data using a customized pipeline (https://github.com/kylegontjes/ISScreener). Trimmomatic v0.39 was used for trimming adapter sequences and low-quality bases. Filtered reads were mapped to the matching K capsule operon reference sequence obtained from Kaptive version 3.0.0b6 using the Burrows-Wheeler short-read aligner (bwa v0.7.17). Samtools v1.19 converted output to a sorted bam file. PCR duplicates were removed using picard v4.1.3. panISa v0.1.7 was run on these alignments to identify potential insertion sequences. The script ISFinder_search.py from panISa package was used to identify IS family from the ISFinder.

Kaptive and Kleborate

For Kp4819, KPPR1, and NJST258_2 WT strains and acapsular isolates the capsule and LPS operon types and virulence scores were generated using Kleborate v3.1.2[3,4]. Capsule typing of all 245 rectal isolates was performed using Kleborate v3.1.2[4].

Curation of Phylogenetic Tree

The assemblies and annotation files of the 245 rectal isolates were acquired from NCBI's RefSeq database (BioProject: PRJNA789565) using NCBI Datasets[5]. The R package, *cognac*, was used to generate an amino acid alignment of 3101 core genes, totaling 990763 amino acids, with 93463 polymorphic sites[6]. Orthologous genes were identified using CD-HIT with a 70% identity threshold and 80% coverage threshold. Core genes were defined as single-copy genes present in 99% of genomes. Phylogenetic reconstruction was performed on marker genes, defined as having at least one variant in the sequence, using the neighbor-joining tree estimation algorithm, as implemented in the R package *ape*[6]. Each isolate's species and sequence type was identified using Kleborate version 3.1.2[4]. The isolate's species, sequence type, case status and the presence of insertion sequence elements in the K-locus were overlaid on the phylogenetic tree using ggtree version 3.12.0[7,8]. Sequence types without an insertion sequence in the K-locus were labeled 'Other'.

Detection of Large Deletion Events

Read mapping of the evolved strains to their ancestral sequence was leveraged to identify large deletions in the K-locus, O-locus, and *rfaH*. Kleborate version 3.1.2 was used to determine each reference genomes' best-matching K- and O-locus[9]. BLAST version 2.15.0 identified the genomic coordinates of these loci for downstream analysis[10]. Coverage data was generated on bam files using the Genome Analysis Toolkit's DepthOfCoverage command[11]. Run length encoding was performed, using the base R function *rle*, to identify consecutive nucleotides with no read mapping. Deletions greater or equal to 100 nucleotides were reported.

References

1. Datsenko, K. A. & Wanner, B. L. One-step inactivation of chromosomal genes in *Escherichia coli* K-12 using PCR products. *PNAS* **97**, 6640–6645 (2000).
2. Wishart, D. S. *et al.* PHASTEST: faster than PHASTER, better than PHAST. *Nucleic Acids Research* **51**, W443–W450 (2023).
3. Wick, R. R., Heinz, E., Holt, K. E. & Wyres, K. L. Kaptive Web: User-Friendly Capsule and Lipopolysaccharide Serotype Prediction for *Klebsiella* Genomes. *Journal of Clinical Microbiology* **56**, 10.1128/jcm.00197-18 (2018).
4. Lam, M. M. C. *et al.* A genomic surveillance framework and genotyping tool for *Klebsiella pneumoniae* and its related species complex. *Nat Commun* **12**, 4188 (2021).
5. O'Leary, N. A. *et al.* Exploring and retrieving sequence and metadata for species across the tree of life with NCBI Datasets. *Sci Data* **11**, 732 (2024).
6. Crawford, R. D. & Snitkin, E. S. cognac: rapid generation of concatenated gene alignments for phylogenetic inference from large, bacterial whole genome sequencing datasets. *BMC Bioinformatics* **22**, 70 (2021).
7. Vornhagen, J. *et al.* Combined comparative genomics and clinical modeling reveals plasmid-encoded genes are independently associated with *Klebsiella* infection. *Nat Commun* **13**, 4459 (2022).
8. Yu, G., Smith, D. K., Zhu, H., Guan, Y. & Lam, T. T.-Y. ggtree: an r package for visualization and annotation of phylogenetic trees with their covariates and other associated data. *Methods in Ecology and Evolution* **8**, 28–36 (2017).
9. Lam, M. M. C., Wick, R. R., Judd, L. M., Holt, K. E. & Wyres, K. L. Kaptive 2.0: updated capsule and lipopolysaccharide locus typing for the *Klebsiella pneumoniae* species complex. *Microb Genom* **8**, 000800 (2022).

10. Altschul, S. F., Gish, W., Miller, W., Myers, E. W. & Lipman, D. J. Basic local alignment search tool. *Journal of Molecular Biology* **215**, 403–410 (1990).

11. McKenna, A. *et al.* The Genome Analysis Toolkit: A MapReduce framework for analyzing next-generation DNA sequencing data. *Genome Res.* **20**, 1297–1303 (2010).
